# ﻿Available names for *Rangifer* (Mammalia, Artiodactyla, Cervidae) species and subspecies

**DOI:** 10.3897/zookeys.1119.80233

**Published:** 2022-08-26

**Authors:** Lee E. Harding

**Affiliations:** 1 2339 Sumpter Drive, Coquitlam, BC, V3J 6Y3, Coquitlam, Canada unaffiliated Coquitlam Canada

**Keywords:** caribou, Reindeer, systematics, taxonomy

## Abstract

Advancements in molecular and phylogenetic analysis have revealed the need for greater taxonomic resolution since *Rangifer* (Reindeer and caribou: Cervidae) was last revised in 1961. Recent literature shows that many of the subspecies and several species synonymised out of existence are, in fact, valid, some names have been misapplied, and new subspecies-level clades are in need of description. This paper reviews available names for recently defined ecotypes of reindeer and caribou in compliance with ICZN rules for zoological nomenclature.

## ﻿Introduction

**Figure 1.**
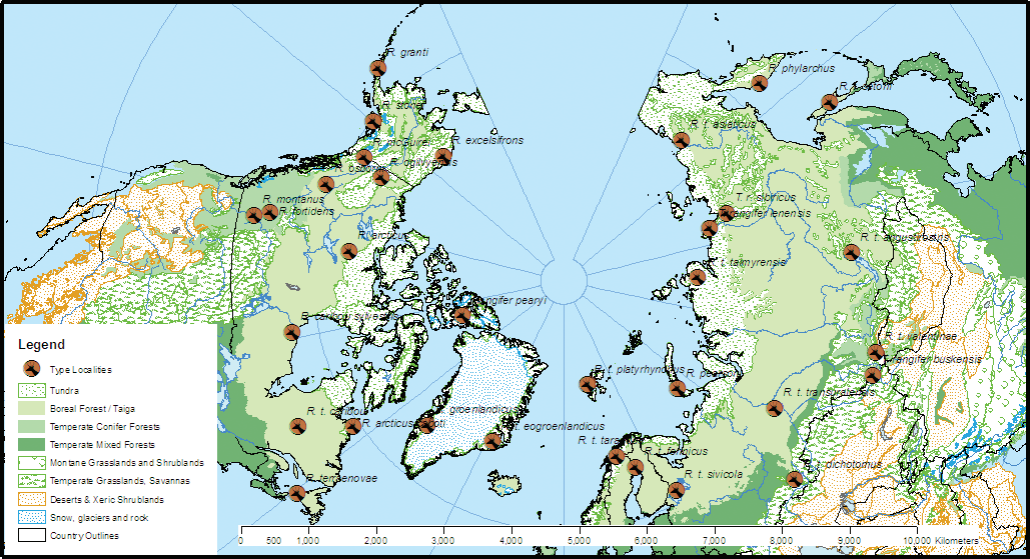
All *Rangifer* type localities overlaid on WWF terrestrial biomes (Olson et al. 2001).

Eighteen *Rangifer* species or subspecies have been named in North America; 31 in Europe and Asia (Fig. 1; see Suppl. material 1: Synonymy). The Mammal Diversity Database, a digital, publicly accessible, and regularly updated list of all mammalian species (Burgin et al. 2018), lists 51 synonyms of *Rangifertarandus* L., 1758. Although many were unjustified by evolving standards and definitions of species (e.g., Mayr 1963; Masters and Spencer 1989; Nixon and Wheeler 1990), the DNA revolution has revealed diversity at the species and subspecies levels that is not reflected in current classifications.

**Figure 2.**
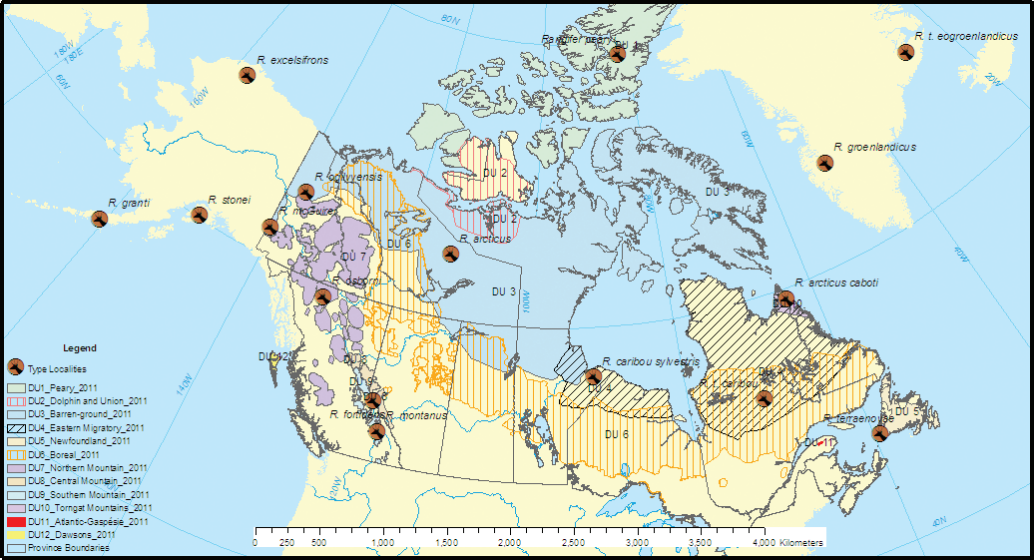
COSEWIC designated units of caribou overlain with *Rangifer* type localities in North America.

*Rangifer* species and subspecies are called reindeer in Eurasia and caribou in North America. As species concepts evolved, Ellerman and Morrison-Scott (1951) lumped all of the Eurasian species into one and implied the same for North America by giving the distribution of *Rangifertarandus* as "Arctic regions of Old and New Worlds … Arctic regions of North America, Greenland included.” Banfield (1961) accepted this for the species and further lumped subspecies, leaving just four in Canada or six, counting the extinct Dawson caribou of Haida Gwai and the introduced Eurasian reindeer. Banfield reduced the caribou of Alaska and Yukon, which formerly had six species or subspecies (Fig. 2), to one subspecies, *R.t.granti*. Now even *granti* is gone, subsumed into *groenlandicus*.

McTaggert Cowan (1962) objected immediately, finding that Banfield (1961), having lumped valid subspecies into one another but still needing to distinguish obviously different kinds of caribou, created a sub-subspecific category, "demes”, a concept not applicable in this context; had used inappropriate statistical methods to summarise and compare morphological data to define subspecies and "demes”; did not provide quantitative characteristics differentiating between adjoining subspecies or demes; failed to show how the "graphs and tables … support …the conclusions drawn”; and even "exceeded his quota” on spelling and grammar mistakes.

Many ungulate taxonomists (e.g., Corbet 1978; Gauthier and Farnell 1986; Groves and Grubb 1987, 2011; Geist 1998, 2007; Mallory and Hillis 1998; Couturier et al. 2010) agreed that Banfield’s (1961) scheme did not reflect subspecies diversity based on morphological measurements.

Despite clear morphological distinctions and profound ecological and behavioural differences, Canadian biologists have felt taxonomically bound by Banfield’s (1961) inadequate and obsolete classification, perhaps in part because he entrenched it further in ‘Mammals of Canada’ (Banfield 1974). Needing, for management and conservation, to continue distinguishing these different caribou, Canadian biologists began referring to distinctive populations as "ecotypes” (e.g., Nagorsen 1990). Since ecotypes are not phylogenetically based, however, they cannot substitute for taxonomy.

Not so elsewhere: in their seminal works, ‘Mammalian Species of the World’, Wilson and Reeder (2005) followed by Wilson and Mittermeier (2011), revised the subspecies under the circumpolar *Rangifertarandus*, citing Markov et al. (1994) and Geist (2007), to validate three subspecies in North America and eight in Eurasia that Banfield (1961) had synonymised.

Molecular analyses are showing how discrete, diagnosable caribou populations differ from Banfield’s (1961) taxonomy. COSEWIC (2011), noting that Banfield (1961) "is out-of-date with respect to current science and does not capture the variability of caribou across their range in Canada,” defined 12 "designatable units” (DU: Fig. 2) for conservation and management. This designation, an adaptation of "evolutionary significant units” (cf. Waples 1995), makes each a "wildlife species” within the meaning of the Species at Risk Act, which provides for recognition of intraspecific populations (cf. Harding 2020). DUs were based on biological, morphological, ecological and, importantly, genetic data; their ranges largely paralleled those of currently accepted or previously named subspecies (or species), without naming them as such, and with new English names.

The purpose of this paper is to review available Latin and English names for distinct reindeer and caribou populations identified by molecular data.

## ﻿Materials and methods

This review is based on both historic and recent literature. Maps were made using ArcMAP GIS layers (ESRI 2004) including the World Wildlife Fund terrestrial biomes (Olson et al. 2001). There was no research on live animals.

## ﻿Results

### ﻿Caribou evolution

*Rangifer* originated in the early Pleistocene, a 2+ million-year period of multiple glacier advances and retreats. Several named *Rangifer* fossils in Eurasia and North America predate the evolution of *Rangifertarandus* sensu lato (Croitor 2018). *Rangiferconstantini* Flerov, 1934, for example, was described from late Pleistocene deposits throughout central Eurasia. Despite its adaptations for open-landscape grazing, it was not adapted to very cold Arctic conditions. Archaeologists distinguish modern tundra reindeer from their ancestors, in part, on the basis of the shape of their nasal bones:

"*Unlike modern reindeer, the volume of nasal cavity of*R.tarandusconstantini*is rather small indicating that the Paleolithic reindeer did not evolve yet adaptations to cold air breathing (Flerov 1952). The function of increased nasal cavity is air warming and moistening before its entrance to the trachea and lungs. Nasal cavity is correlated with muzzle breadth and the maximal volume of nasal cavity is recorded in modern arctic reindeer (Croitor 2018).*”

The oldest North American *Rangifer* fossil is from Yukon, 1.6 million years before present (BP) (Harington 2011). A fossil skull fragment from Süßenborn, Germany, *R.arcticusstadelmanni* Kahlke, 1963, with "rather thin and cylinder-shaped” (Kahlke 1963) antlers (this refers to a fundamental difference between "arcticus-type” and woodland caribou antlers, which are flattened in cross-section), dated to the middle Pleistocene (Günz) period, 680,000 to 620,000 BP (Croitor 2010). *Rangifer* fossils become increasingly frequent in circumpolar deposits beginning with the Riss glaciations (Banfield 1961), the second youngest of the Pleistocene epoch, roughly 300,000–130,000 BP. By the 4-Würm period (110,000–70,000 to 12,000–10,000 BP) its European range was extensive (Kurtén 1968), supplying a major food source for prehistoric Europeans.

**Figure 3.**
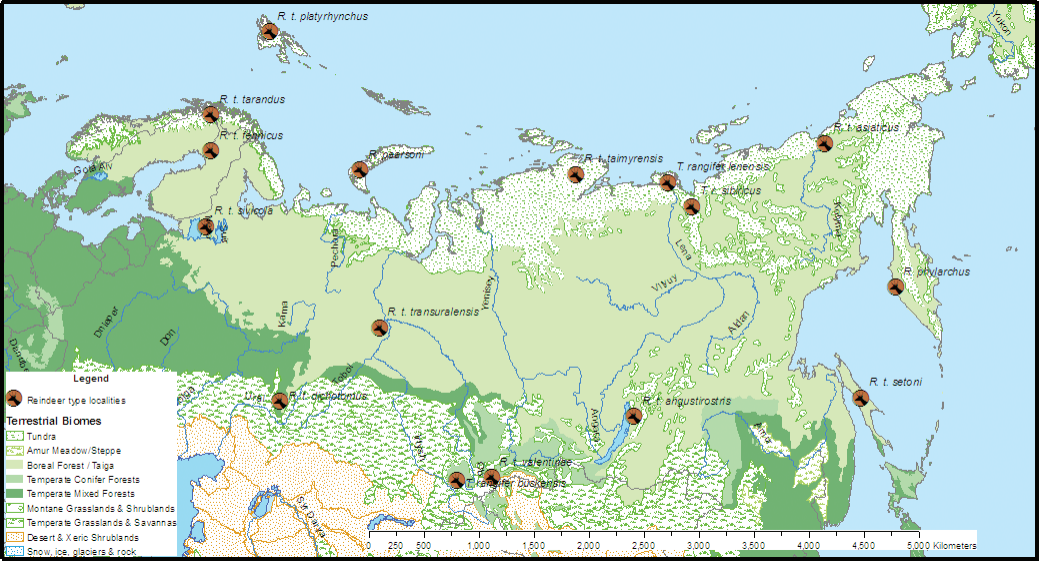
*Rangifer* type localities in Eurasia overlaid on WWF terrestrial biomes (Olson et al. 2001).

Geist (1998) notes that European prehistoric cave paintings represent both tundra and forest forms, the latter either *R.t.fennicus* or *R.t.angustirostris*, an eastern Siberian forest form (Fig. 3). DNA analysis showed that people independently domesticated reindeer at least twice, both from tundra forms after the last glacial maximum (LGM), in Fennoscandia and western Russia, and possibly also eastern Russia (Røed et al. 2008; Weldenegodguad et al. 2020).

North American fossils outside of Beringia that predate the LGM are of Rancholabrean age (240,000–11,000 years BP) and occur along the fringes of the Rocky Mountain and Laurentide ice sheets as far south as northern Alabama; and in Sangamonian deposits (~ 100,000 years BP) from western Canada (Geist 1998 and references therein).

### ﻿Subspecies and ecotypes

*Rangifertarandus* subspecies accepted by the American Society of Mammologists and referenced to Wilson and Mittermeier (2011) are: Eurasia (Fig. 3): *tarandus*, *buskensis*, *fennicus*, *pearsoni*, *phylarchus*, *platyrhynchus* and *sibiricus*; and North America (Fig. 2): *caboti*, *caribou*, *dawsoni*, *groenlandicus*, *osborni*, *pearyi*, and *terraenovae*. Authorities of all taxa cited in the text are given in Suppl. material 1: Synonymy.

#### Europe

Russian scholars (e.g., Kharzinova et al. 2018; Mizin et al. 2018; Rozhkov et al. 2020; Vasilchenko et al. 2020) recognise 4–8 subspecies within Russia (Fig. 3): those mentioned above plus *angustirostris*. Of these, *fennicus*, *valentinae*, *angustrostris*, and *phylarchus* are forest reindeer and are larger, longer-legged, and darker and have shorter, heavier, and more branched antlers than tundra reindeer (Baskin 1986; Rozhkov et al. 2020). Although Wilson and Reeder (2005), followed by Wilson and Mittermeier (2011), chose *R.t.buskensis* Millais, 1915, as a senior synonym for *R.t.valentinae*, Millais (1915a) is not a valid taxonomic authority (see Discussion).

#### Eurasian Tundra reindeer

Because of Banfield’s (1961) lumping, Western scholars have often not distinguished between true *R.t.tarandus* of the western European mountains, and the far more widespread *R.t.sibiricus*. For this reason, papers on genetic diversity must be read carefully to determine the provenance of the specimens.

Although Eurasian tundra reindeer and North American barren-ground caribou are distinguishable by different allele frequencies at several loci, they have low genetic differentiation (Cronin et al. 2005). They have diagnostically different pelage patterns and other morphological differences (see Geist 1998 for descriptions).

The "mountain reindeer” of Norway (and formerly Sweden and Finland) are tundra reindeer (*R.t.tarandus*) that have adapted to high-elevation alpine tundra with snow characteristics similar to Arctic tundra: hard-packed, shallow snow that they can paw through to reach terrestrial lichens. The haplotype composition of reindeer from southern Norway is similar to, but "substantially genetically different from” that of the tundra reindeer from western Russia, *R.t.sibiricus* (von Schreber, 1784) (Baranova et al. 2012).

*Rangifert.sibiricus* includes 19 herds, named for their calving grounds, from Arkhangelsk in European Russia to Chukotka, Siberia (Mizin 2018; Rozhkov et al. 2020). Although domestic reindeer descended from tundra types, there is a "clear genetic differentiation between domestic and wild reindeer populations” with ~ 6% introgression by wild reindeer into domestic clades and none the other way (Kharzinova et al. 2018; Rozhkov et al. 2020). There is little genetic difference among wild tundra populations of *R.t.sibiricus* in Taymyr, northern Yakutia, and Chukotka (Kharzinova et al. 2018).

Based on mtDNA, wild reindeer in Genhe, north of the Greater Khingan Mountains in Heiliongjiang, China (Temperate Coniferous Forest zone, Fig. 3), are of Beringian-Eurasian lineage semi-domesticated by the Ewenki people that lost their migratory habits, not forest reindeer as previously supposed (Wang et al. 2019; Ju et al. 2020).

#### Svalbard reindeer

Despite Lydekker’s (1915) attempt to bring it under *R.tarandus*, Sokolov (1937, 1963) insisted that its skull shape, especially the rostrum, and the dentition, were different enough to maintain *R.platyrhynchus* Lönnberg, 1909 as a species. Svalbard (and the extinct east Greenland and Peary’s) caribou derived from ancient Beringian-Eurasian pre-glacial populations, based on shared mtDNA haplotypes, but evolved in separate refugia during the LGM (Flagstad and Røed 2003; Kvie et al. 2016). Svalbard reindeer (with West Greenland caribou; see below) are the most genetically distinct of all *Rangifer* subspecies (average genetic differentiation [Fixation Index, F_ST_] 41% to all other reindeer and caribou); they are not, however, closely related to each other, with a 69% genetic differentiation between them (Yannic et al. 2013). The large genetic differentiation qualifies Svalbard reindeer as *R.platyrhynchus* Lönnberg, 1909 (Miller 1912a).

#### Russian Arctic archipelago

Based on mtDNA control region sequences, reindeer of the High Arctic archipelagos of Franz Josef Land and Novaya Zemlya, *R.t.pearsoni* Lydekker, 1902, descended from wild tundra reindeer from the Eurasian mainland after the LGM, but before humans could have brought domesticated reindeer (Kvie et al. 2016). Reindeer occupied Franz Josef Land briefly, having migrated there after the Holocene climatic optimum (~ 6,000–4,500 years ago) when the climate again became colder and the sea‐ice more persistent, and became extinct historically (Mizin et al. 2018). Novaya Zemlya reindeer occupied the island only 7,000–5,000 years ago and were described by their distinctive phenotypical appearance (Lydekker 1902); however, today’s population is mostly feral domestic reindeer, the endemic *R.t.pearsoni* having either not survived or the few remaining individuals mixed with domestic reindeer (Mizin et al. 2018 and references therein).

#### Kamchatka reindeer

The Okhotsk or Kamchatka reindeer, *R.t.phylarchus* Hollister, 1912, has pelage patterns and antler formation more like Canadian barren-ground caribou than other Eurasian subspecies, prompting Geist (1998) to conclude that, "This is no reindeer, but a caribou.” It probably dispersed from Beringia in the late Pleistocene in a "second radiation into Siberia”, after Canadian and Eurasian forms had evolved distinctive patterns and adaptations (Geist 1998). Rozhkov et al. (2020) showed that wild reindeer from Kamchatka cluster separately from those living west of the Sea of Okhotsk, which are indistinguishable genetically from the Jano-Indigirka, East-Siberian taiga, and Chukotka populations of *R.t.sibiricus* (von Schreber, 1784). The range of Kamchatka reindeer therefore should be restricted to the Kamchatka Peninsula.

#### Forest reindeer

Finnish or European forest reindeer, *R.t.fennicus* Lönnberg, 1909, was described from Finnish Lapland (Fig. 3). Ellerman and Morrison-Scott (1951) synonymised it with the Kerelian forest reindeer *R.t.silvicola* Hilzheimer, 1936 and Siberian forest forms *R.t.transuralensis* Hilzheimer, 1936, *R.t.dichotomus* Hilzheimer 1936, and *R.t.angustirostris* Flerov, 1932 as junior synonyms. That it is considerably larger than *R.t.tarandus* "can hardly be due to nutritional factors alone”; it also has:

"*significantly longer legs...[that] are an important adaptation to taiga conditions, where the snow cover is usually deep and soft. The mountain types* [R.t.tarandus*in Norway] have evolved in areas with hard-packed tundra snow, and consequently the semi-domestic reindeer have difficulty surviving in coniferous forests, especially in winters with deep, soft snow (Nieminen and Helle 1980).*”

*Rangifert.fennicus* has statistically significant cranial differences from tundra reindeer, particularly its arched nasal bones (flattened in tundra reindeer: Banfield 1961). Its antlers are "…reminiscent of barren-ground antlers, but with oval beams and a bez tine set well above the brow tine…shaped like tundra-type antlers but more massive and show some palmation” (Geist 1998). Its pelage pattern is similar (see Geist 1998 for descriptions).

*Rangifert.fennicus* evolved in isolation from the tundra type in a separate western European refugium and adapted to forest environments; it shares no mtDNA haplotypes with any North American caribou (Flagstad and Røed 2003; Røed 2005).

Between wild tundra and taiga reindeer (subspecies not stated; presumably *R.t.fennicus* sensu lato) pairwise F_ST_ values, using a "genome-wide bovine SNP genotyping array”, averaged 3.8%–9.4%, "consistent with their morphological and ecological differences” (Kharzinova et al. 2018). Weldenegodguad et al. (2020), based on microsatellites, also found that Finnish reindeer clustered separately from all other ecotypes (Eurasia tundra, Alaska, and Svalbard). Genetic distances (see Suppl. material 2: Genetic distance) and differences in morphology and ecology between *fennicus* and *tarandus* suggest that the former should be returned to full species status, with subspecies *R.fennicusfennicus*, *R.f.valentinae*, and possibly *R.f.angustirostris*.

*Rangifert.angustirostris*, the East Siberian forest reindeer, currently numbers ~ 1,000 animals, distributed east of Lake Baikal (Mizin 2018). Its status, whether more allied to *fennicus* than to *sibiricus*, is best "left in doubt until data on its genetics become available” (Rozhkov et al. 2020).

Croitor (2010) hypothesised that *R.t.fennicus* evolved from *Cervusguettardi* Desmarest, 1822, a reindeer that adapted to forest habitats in western Europe as forests expanded during an interglacial period before the LGM (the Würmian or Weichsel glaciation); *guettardi* was later replaced by *R.constantini*, a more evolved tundra form (cf. Baranova et al. 2016), in a second immigration 19,000–20,000 years ago when the LGM turned its forest habitats into tundra, while *fennicus* survived in isolation in south-western Europe. If correct, *fennicus* does not share a common ancestor with *R.tarandus* and cannot be conspecific. Its name would be *Rangiferfennicus* Lönnberg (Miller 1912a).

Wild reindeer from Murmansk/Kola Peninsula are forest reindeer, *R.t.fennicus*, sharing a clade with those from Karelia and Arkhangelsk; these share two haplotypes with domestic reindeer from the same regions, but show only a low incidence of hybridisation, indicating ancient introgression (Baranova et al. 2016; Korolev et al. 2017; Vasilchenko et al. 2020).

The Altai-Sayan forest reindeer (*Rangifertarandusvalentinae* Flerov, 1933) is a montane form whose ecology parallels that of British Columbia’s mountain caribou (see below). It migrates altitudinally in dense coniferous forests at elevations of 400–1,500 m, where snow cover is 130–250 cm, and forages arboreal lichens in winter (Baskin 1986). Its mating system also is similar to that of British Columbia mountain caribou: males guard 3–5 females during rutting season and calving is dispersed in alpine habitats (Shaposhnikov 1955, cited by Sobansky 1976). It has a unique mitochondrial genome and shows no signs of introgression of domestic reindeer mtDNA (Vasilchenko et al. 2020). Its genetic differentiation (using a genome-wide genotyping array to compare single nucleotide polymorphism, SNP, markers) from three populations of putative *R.t.sibiricus* is F_ST_ = 3.1%–3.5% (Kharzinova et al. 2018). See Geist (1998) for descriptions of pelage patterns.

Interestingly, the two forest forms, *fennicus* and *valentinae*, cluster together as sister clades, based on mtDNA haplotypes, even though separated by 3,800 km and with the East European Taiga population of *sibiricus* between them; and these two form a sister clade with a Siberian taiga population of *sibiricus* to the exclusion of *tarandus*, *pearsoni*, and *phylarchus* (Rozhkov et al. 2020). This qualifies them as *R.fennicusvalentinae*. Davydov et al. (2007) also united *valentinae* with *fennicus* as closely-related subspecies that clustered apart from tundra and Arctic island forms in Eurasia and North America.

#### North America

Early genetic analyses showed two major lineages of caribou in North America: migratory barren-ground caribou, whose ancestors survived the LGM in Beringia, that calve on the tundra and migrate in winter to boreal forest; and a non-migratory, exclusively forest clade whose ancestors persisted south of the ice-sheets that covered northern North America and the western cordillera (Courtois et al. 2003; Flagstad and Røed 2003; Zittlau 2004; Cronin et al. 2005). COSEWIC (2011) and others refer to these as the BEL (Beringian-Eurasian) and NAL (North American) lineages, respectively.

Currently recognised Canadian BEL barren-ground caribou subspecies are *R.t.groenlandicus* sensu lato of the mainland tundra, *R.t.caboti* of Labrador, *R.t.osborni* of the northern cordillera, *R.t.pearyi* of the High Arctic, and the extinct insular *R.t.dawsoni* (Wilson and Mittermeier 2011). However, Banfield (1961) erred, both in failing to recognise the species-level separation of *R.tarandus* from *R.arcticus*, and in assigning the subspecies name *groenlandicus* to mainland barren-ground caribou, as discussed below. Its proper name is *R.arcticus* Richardson, 1829 (Allen 1942).

#### Western montane ecotypes

All three western montane ecotypes (Osborn’s caribou, Rocky Mountain caribou and Selkirk caribou: Fig. 2) are of BEL ancestry, but are deeply divergent genetically and ecologically, having split from barren-ground caribou some 60,550 years ago in the Illinois-Wisconsin interglacial; each is a different combination of separate BEL lineages (Klütsch et al. 2012; Polfus et al. 2017; Taylor et al. 2021).

The "southern group of the Southern Mountain population of Woodland caribou”, *R.taranduscaribou* (cf. COSEWIC 2014) was originally described as the Mountain or Selkirk caribou, *R.montanus* Seton-Thompson, 1899; the Central Mountain population was Rocky Mountain caribou, *R.fortidens* Hollister, 1912; and the Northern Mountain population was Osborn’s caribou, *R.osborni* Allen, 1902. These scientists distinguished the three mountain types based on quantitative differences in dentition, skeletal and antler measurements, pelage colour and size, as well as ecology.

Anderson (1946) concurred with Jacobi (1931) in retaining the subspecies designations of all three western montane ecotypes under Arctic caribou: *R.arcticusosborni*, *R.arcticusmontanus*, and *R.arcticusfortidens*.

When Ellerman and Morrison-Scott (1951) revised *Rangifer* into a single species, the Eurasian name, *R.tarandus* taking priority, Selkirk caribou became *R.t.montanus*, and Osborn’s caribou, *R.t.osborni* (McTaggart Cowan and Guiguet 1956). Significantly, although Banfield (1961) acknowledged that his measurements showed Osborn’s and Selkirk caribou as morphologically separate from each other and from barren-ground caribou and woodland caribou, he still lumped them with *R.t.caribou* Gmelin, 1788. Nagorsen (1990), then Curator of Mammals at the Royal British Columbia Museum, objected: "...these two morphs [Mountain and Osborn’s] exhibit some differences in size, antler morphology and pelage colour… a modern study of geographic variation … is needed to resolve the systematics of woodland-mountain caribou”.

Geist (1991) maintained the separation of *montanus* from *osborni* on the basis of size, pelage patters and colour, *montanus* being smaller and darker. Osborn’s caribou, currently recognised as *R.t.osborni* (Wilson and Mittermeier 2011), therefore reverts to *R.a.osborni*Jacobi (1931).

Serrouya et al. (2012), Harding et al. (2020) and others called the Selkirk caribou "deep-snow mountain caribou”, because, uniquely, they winter high on the mountains where they walk on top of a 2–5 m deep snowpack to forage arboreal lichens. The name, *Rangiferarcticusmontanus* Seton-Thompson, 1899 (Jacobi 1931) is available; or *Rangifermontanus* Seton-Thompson, 1899, as Murie (1935) insisted and as its genetic distance from others (see above and Suppl. material 2: Genetic distance) suggests.

Rocky Mountain caribou, or the Central Mountain population DU8 per COSEWIC (2011), occupy the east slope of the Rocky Mountains (Fig. 2) where the continental climate results in light, shallow snow in which they forage terrestrial lichens in winter. They average 55 km horizontal migration to forested winter ranges, a little less than Osborn’s caribou and far more than "sedentary” boreal woodland caribou (Theoret et al. 2022). They are mountain caribou that have hybridised in ancient times with woodland caribou, with which they share haplotypes (McDevitt et al. 2009; Taylor et al. 2021). The name *R.a.fortidens* Hollister, 1912 (Jacobi 1931) is available and appropriate.

#### Haida Gwai

A caribou antler from Haida Gwaii, British Columbia was dated to ~ 43,200 years BP in the mid-Wisconsin Olympia Interglacial (Mathewes et al. 2019). More recent (4,000–6,000 BP) bones of the extinct, insular Dawson caribou, originally *R.dawsoni* Seton-Thompson, 1900, were similar to barren-ground caribou but smaller, evidence of island dwarfism (Byun et al. 2002 and references therein). Evidently, they evolved in a coastal refugium after the LGM when rising sea levels isolated them. Byun et al. (2002), using molecular and ancient DNA techniques, were able to sequence a short fragment of the mtDNA from remains of Dawson caribou. Their results allied them phylogenetically with the caribou on the adjacent mainland (which at the time were thought to be *R.t.caribou*, but which are now known to be BEL lineage *R.a.osborni*) and a little less closely to Alaska barren-ground caribou (see Suppl. material 2: Genetic distance).

#### Alaska-Yukon

In Alaska, of 13–32 caribou herds that have been recognised, including four that overlap with Yukon (Hemming 1971; Valkenburg 1998), four (Porcupine, Central Arctic, Teshekpuk, and Western Arctic) are small, phenotypically barren-ground caribou with long migrations and aggregated, tundra calving grounds (Prichard et al. 2020), while a fifth (Steese-Fortymile) is intermediate in form and behaviour (Hemming 1971; Gauthier and Farnell 1986; Valkenburg 1998; Mager et al. 2014).

Allen (1902) described Grant’s caribou, *R.granti* Allen, 1902, of the Alaska Peninsula and archipelago (Fig. 2), noting that it was "not closely related to *R.stonei* of the Kenai Peninsula, from which it differs not only in its very much smaller size, but in important cranial characters and in [pale] coloration.” It remained a species or subspecies (see Suppl. material 1: Synonymy) until Banfield (1961) erroneously brought all other Alaska caribou under it as junior synonyms, thus expanding its range to the whole state and northern Yukon. Youngman (1975) began a trend to replace *granti* with *R.t.groenlandicus* sensu lato. Because Geist (1998) could find no morphological features to distinguish Alaskan from Canadian barren-ground caribou, *granti* was not accepted by Wilson and Reeder (2005) and Wilson and Mittermeier (2011). Caribou geneticists agree that they are barely distinguishable (e.g., Cronin et al. 2005; Yannic et al. 2018). As originally described, however, *granti* survives (see below).

Murie (1935) brought *Rangiferexcelsifrons* Hollister, 1912, *Rangifermcguirei* Figgins, 1919, and *Rangiferogilviensis* Millais, 1915, under *R.stonei* Allen, 1901 (Fig. 2). Stone’s caribou was said to range from the Kenai and Alaska Peninsulas to western Yukon "and more sparingly to the eastward” (Murie 1935): a large, dark caribou with a well-developed white fringe on the throat and "antlers large and rangy, of the *arcticus* type, but heavier”. The former *R.ogilviensis* (Millais, 1915b) is the Porcupine Herd (named for a river that flows from Yukon into Alaska) of barren-ground caribou that winters mainly in the Ogilvie Mountains, Yukon, and calves on the Alaska-Yukon coastal plain. Their migratory, rutting and calving-aggregation behaviours and small size (consistently smaller than mountain caribou to the south and west: Gauthier and Farnell 1986) reveal their barren-ground identity.

In Alaska, some two dozen herds are genetically, morphologically (larger and darker than barren-ground caribou: Gauthier and Farnell 1986) and ecologically similar to the western Canada montane forms (Mager et al. 2014 sampled 20 of the 26 currently recognised herds). Nevertheless, they clustered clearly into two groups at *K* = 2, one of which encloses the Alaska Peninsula type locality of *R.granti* (Fig. 2). Colson et al. (2014) found a lack of dispersal or introgression from adjacent ecotypes, suggesting reproductive isolation of the Alaska Peninsula/archipelago cluster. Yannic et al. (2018) confirmed the genetic distinctiveness of this ecotype, which had been previously found to differ morphologically as well (cf. Murie 1935; see Suppl. material 1: Synonymy). Thus, *R.a.granti* is rediscovered, its range restricted (as originally: Allen 1902) to the Alaska Peninsula and archipelago.

At *K* = 4, six "mainland” (i.e., not peninsula/island) herds, all geographically small, isolated mountain herds, "appeared relatively discrete with > 0.50 population assignment to one cluster, rather than several [and] had high pairwise differentiation” (Mager et al. 2014). One of these, the Chisana herd, which spans the Alaska-Yukon border, contains the type locality (Fig. 2) of *Rangifermcguirei* Figgins, 1919. Figgins (1919) found differences, which he thought sufficient to name a new species, in the pelage, cranial and dental features, and antler formation of six specimens of Chisana caribou, compared to the same morphological characters in *osborni* and *stonei*. Murie (1935), followed by Anderson (1946), synonymised *mcguirei* with *stonei* because "No part of the original description would distinguish the type specimen from *R.arcticusstonei*. Furthermore, the type locality lies squarely in the path of migration of the large herd of *stonei*, the principal herd of Alaska-Yukon caribou, at a point where hundreds of thousands pass through each year during the rutting season.”

This reasoning is flawed because none of these mountain herds migrate long distances: rather, each migrates altitudinally if at all (most winter in alpine tundra where wind clears snow from the terrestrial lichens) and maintains separate alpine rutting and calving areas. Murie (1935) no doubt had observed the Steese-Fortymile herd on migration. Nevertheless, Osgood (1909) and Murie (1935) were prescient in bringing all of these under *R.arcticus*.

Likewise, 16 southern Yukon and northern British Columbia herds, 15 of them currently identified as *R.t.osborni*, clustered into four groups based on microsatellite DNA analysis and three based on mtDNA (Kuhn et al. 2010). Haplotypes of the migratory Steese-Fortymile herd (the others are sedentary), were spread throughout the others, suggesting it as ancestral to all with perhaps occasional introgression.

The clustering pattern described above (Kuhn et al. 2010; Mager et al. 2014) argues for restoring the subspecies name, *R.a.stonei* Allen 1901 (Murie 1935), to Chisana and all other interior Alaska mountain caribou that cluster together and apart from *osborni*, *granti* sensu stricto, and Alaskan barren-ground caribou, including Steese-Fortymile, as subspecies of *R.arcticus*. This is another case of the pre-Banfield taxonomy being confirmed by genetic data.

Introductions of *R.t.tarandus* sensu lato and *R.t.sibiricus* into Alaska and thence to Nunavut were detailed by Anderson (1946). Some interbreeding between reindeer and wild caribou in Alaska has been documented, but with very little introgression in either direction, probably because of low fitness of hybrid animals in the wild; relatively little crossbreeding has been observed when the two have been in captivity together (Cronin et al. 2006).

#### Barren-ground Caribou

Banfield (1961) unjustifiably renamed *R.arcticus* as *R.t.groenlandicus* (see below). It has seven recognised herds on mainland Canada, defined by calving grounds, and extends into Alaska. Barren-ground caribou are smaller and paler than woodland caribou, but have longer, thinner antlers that, in males, sweep back, up and forward, main beams reaching > 135 cm with multiple tines at the top, often palmated (see Geist 1998 for diagnostic features). Its beams are round in cross-section vs. flattened in woodland caribou.

Anderson (1913) described the migration of the Dolphin and Union herd, DU2 (Fig. 2), across Dolphin and Union Straits from Victoria Island to the mainland and back. He also briefly described its diagnostic pelage pattern and cranial morphology (e.g., "...The heads of these Caribou appeared to be much shorter than those of the Great Bear Lake Caribou, with a noticeable fullness or convexity between forehead and nose”). He intended to name a new form of caribou and to select a type specimen from among the 84 he collected in 1911 and sent to the American Museum of Natural History; any of mature males AMNH 34431, 34432, and 34435 would be a suitable holotype. He also sent 24 to what is now the Canadian Museum of Nature, but never completed a formal report of his second (1913–1916) expedition (Anderson 1917). Thomas and Everson (1982) later confirmed its unique skeletal features quantitatively. It was long thought to be either a race of Peary caribou or a hybrid between Peary and barren-ground caribou (e.g., Manning 1960) until genetic and other data showed it to be a distinct race of barren-ground caribou (Zittlau 2003; COSEWIC 2004, 2017). Since it was never formally described, there is no available subspecies name for this ecotype.

#### The High Arctic

Peary’s caribou, *R.t.pearyi*, of the Arctic Archipelago except for Baffin Island (Fig. 2), is "clearly most genetically similar to the Canadian barren-ground caribou (*R.t.groenlandicus*) from North West Territories … suggesting common origin of the ancestors of these populations” (Røed 2005). Peary caribou diverged from barren-ground caribou 96,000–185,000 BP and evolved in isolation through two glacial cycles (Klütsch et al. 2017). COSEWIC (2004) restricted its distribution to the High Arctic islands and the Boothia Peninsula, except for most of Victoria Island, based on Harding (2003) and Zittlau (2003). A BEL lineage, it is DU1 (COSEWIC 2011).

#### Baffin Island

Baffin Island caribou comprise insular populations that are geographically and genetically disjunct from both mainland barren-ground and Peary caribou (Fig. 2). It differs from the mainland barren-ground caribou in lacking large-scale migrations and with calving being dispersed rather than aggregated (Jenkins et al. 2012). Its genetic differentiation (pairwise F_ST_ based on 16 microsatellite loci) "is evidence of evolutionary significance and points to Baffin Island caribou as a candidate for consideration as a DU” (Jenkins et al. 2018).The most common allele in Baffin Island caribou is absent or occurs in very low frequencies in other barren-ground caribou populations including the nearby Beverly herd on the adjacent mainland; likewise, the Beverly herd has eight alleles that are absent from the Baffin Island samples, indicating "a large genetic distance” between them (gel electrophoresis: Røed et al. 1991). Baffin Island caribou share one haplotype (C10) with those in Labrador and two each with Dolphin and Union and Bluenose herds of barren-ground caribou (Cronin et al. 2005). The genetic distances (see Suppl. material 2: Genetic distance) and other data suggest at least a subspecies. There is no available name for a Baffin subspecies.

#### Greenland

Small, *pearyi*-sized caribou occupied the ice-free parts of Greenland in the Illinoian-Wisconsin interglacial and through the LGM and early Holocene (Meldgaard 1986). Degerbøl (1957) described *R.t.eogroenlandicus*, which became extinct ~ 1900, from a relict enclave in north-eastern Greenland (Fig. 2). However, Bennike (1988), comparing bones and noting that Peary caribou have been documented crossing Nares Strait to Greenland, doubted that *pearyi* and *eogroenlandicus* were subspecifically distinct. That Peary caribou shared certain mtDNA haplotypes and morphological similarities with it (Kvie et al. 2016) casts further doubt on the validity of *R.t.eogroenlandicus*.

The larger West Greenland caribou is problematic. It is darker than typical *arcticus* and much darker than *pearyi*, resembling woodland caribou in its dark-brown body, with neck and ventral area much lighter (Allen 1908). Allen (1908) gives Greenland caribou average condylobasal length and upper maxillary tooth row metrics, both greater than in mainland *arcticus* and considerably more than in the "little *pearyi*”. He also notes that antlers of Greenland caribou adult males, although within the range of *arcticus* in total length, are "slenderer, less palmated, and more recurved”. Historic and archaeological records show that barren-ground-sized caribou immigrated to West Greenland, possibly from Baffin Island via the Davis Strait, in the middle Holocene (Meldgaard 1986). However, a reconstruction of glacial retreat and caribou advance (Yannic et al. 2013) shows colonisation by NAL lineage caribou more likely.

Greenland caribou, with Svalbard caribou, are the most geographically and genetically isolated ecotypes among all extant caribou (average fixation index 41%: Yannic et al. 2013) based on 16 microsatellite loci. They share low relatedness values with all Canadian caribou (Solmundson et al. in press).

The (West) Greenland caribou is neither of the BEL lineage, from which descend all Eurasian and Canadian tundra reindeer and caribou, nor the NAL lineage of woodland caribou: it clusters outside of the BEL cluster, as do Svalbard reindeer (Yannic et al. 2013). Yannic et al. (2018) were unable to include it in their hierarchical analysis "because of their high genetic differentiation”. It is best left as Linnaeus (1767) originally classified it: *Rangifergroenlandicus* L., 1767 (Miller 1912a).

#### Mackenzie River Valley

Polfus et al. (2017), using nuclear and mtDNA, discovered another distinct "woodland” clade in the Northwest Territories between the Mackenzie River and Great Bear Lake that descends, not from the NAL lineage as other woodland caribou, but from the BEL lineage. They quoted Geist (2007), who, using pelage and antler characteristics and taxonomic inferences, suggested that the mountain and boreal "woodland” caribou north of 60° latitude were more likely "splinter populations of barren ground caribou, which have adapted to a more sessile life-style, increased in body size, and assumed some ‘woodland mannerisms’”. Since the best-supported model of Polfus et al. (2017) shows descent from *R.a.osborni*, it should be considered an ecotype of Osborn’s caribou.

#### Woodland caribou

Gmelin (1788), editing the 13^th^ edition of Linnaeus’ ‘Systema Naturæ’, carried forward Brisson’s (1756) "Le Karibou” as a subspecies, *Cervustarandus γ caribou*. Gmelin (1788) did not name a type locality. Miller 1912b and Lydekker (1915) both gave it as "Eastern Canada” without attribution. Banfield (1963) later designated a neotype and neotype locality at Quebec (City), Province of Quebec, Canada. In Fig. 2, I placed the type locality symbol in the middle of the Province of Quebec.

Historically, most *Rangifer* taxonomists (e.g., Baird 1859; Ross 1861; Allen 1896; Lydekker 1898; Miller 1912b) thought the differences from barren-ground caribou warranted species status, *R.caribou*, based on the larger size, darker colour, different antlers, and sedentary habits (see Suppl. material 1: Synonymy). Lydekker (1915) described woodland caribou antlers as "...stout, flattened, much palmated, and not of excessive length, one of the brow-tines being much expanded, while the other is simple; the bez-tine is also more palmated than in the Scandinavian reindeer.”

Genetic and morphological analyses (e.g., Klütsch et al. 2012; Horn et al. 2018; Yannic et al. 2018) have confirmed the woodland caribou’s distinctiveness and divergence from other ecotypes ~ 357,000 BP (Horn et al. 2018). These differences and its genetic distance from other ecotypes (see Suppl. material 2: Genetic distance) warrant restoration of *Rangifercaribou* Gmelin, 1788 (Baird 1859).

Three haplogroups of woodland caribou are evidence of isolation in three refugia (in the Rocky Mountains, east of the Mississippi, and the Appalachian Mountains) during the LGM, giving rise to two clades of boreal woodland caribou east and west of a "suture zone” in Manitoba (Klütsch et al. 2012). A fourth, coastal refuge was later identified (Wilkerson et al. 2018). Mid-continent clades have a few barren-ground haplotypes, arising after the LGM when the latter rutted far enough south to encounter the former; however, barren-ground caribou do not have woodland caribou haplotypes (McQuade Smith 2009; Yannic et al. 2018).

COSEWIC (2011), based mainly on molecular data, divided Banfield’s (1961) woodland caribou, *R.t.caribou*, into five Designated Units (Fig. 2): Eastern Migratory DU4, Newfoundland DU5, Boreal DU6, Torngat DU10, and Atlantic-Gaspésie DU11, in addition to the three western montane ecotypes discussed above.

Richardson (1829), without specifying a type locality, had described the "western woodland caribou”, *R.t.sylvestris* (Richardson, 1829). He gave its range as west of Hudson’s Bay and James Bay in rocky (Precambrian Shield) habitat, west to Lake Superior and Lake Athabasca. In Fig. 2, I placed the type locality symbol approximately in the middle of the range given by Richardson (1829). He said:

"*Contrary to the practice of the Barren-Ground Caribou, the Woodland variety travels to the southward in the spring. They cross the Nelson and Severn Rivers [in Manitoba and Ontario, respectively] in immense herds in the month of May, pass the summer on the low, marshy shores of James’ Bay, and return to the northward, and … retire more inland in the month of September.*”

Richardson (1829), although one of the greatest Arctic explorers of his era, had little experience with caribou south of the Barrenlands. He did not mention having examined museum specimens back in London, did not figure the animal or give metrics supporting its being "much larger than the Barren-Ground Caribou [and] has smaller horns” and most of his account of *R.t.sylvestris* is hearsay. His description of "immense herds” in migration precludes its application to woodland caribou.

Serrouya et al. (2012), using microsatellite markers, confirmed earlier conclusions (McLoughlin et al. 2004) that the caribou of the woodland ecotype DU6, sampled in Alberta north and south of the Peace River, clustered separately and were genetically distant from each other (average F_ST_ = 5.9% between pairs). Could those north of the Peace River be the newly discovered BEL clade of boreal "woodland” caribou in the Mackenzie Valley (cf. Polfus et al. 2017)?

#### Atlantic-Gaspésie caribou

The Atlantic-Gaspésie caribou (Fig. 2), DU11 (COSEWIC 2011), is an isolated montane ecotype (Pelletier et al. 2019). It is significantly differentiated from Labrador and boreal woodland caribou in Québec, F_ST_ 10.3%–17.2%, based on microsatellite loci (Courtois et al. 2003). Yannic et al. (2018), also based on microsatellite loci, gave its average F_ST_ from all other Canadian populations as 19%. This and its unique ecology (Pelletier et al. 2019; Frenette et al. 2020), warrant at least subspecific distinction. It remains unnamed.

#### Labrador or Ungava caribou

Allen (1914) described the "Barren-ground Caribou of Labrador” from the Ungava Peninsula (Fig. 2) as *Rangiferarcticuscaboti* Allen, 1914. Jacobi (1931) and Anderson (1946) thought it morphologically distinct enough for species status, *R.caboti*. Geist (1998) considered it a diagnostically "distinct form of barren-ground caribou” with antlers of "classical barren-ground form, but with short tines; brow and bez tines very close together; antlers usually widely spread”. Loughrey and Kelsall (1970) called it a migratory form of woodland caribou, *R.c.caboti*, and Cronin et al. (2005) confirmed that Labrador caribou share mtDNA haplotypes and have similar microsatellite allele frequencies to woodland caribou with ancient admixture from barren-ground caribou. There has been little recent introgression (Boulet et al. 2007; Klütsch et al. 2016; Taylor et al. 2021). It shares only one haplotype with barren-ground caribou and that is from the Baffin Island population (Cronin et al. 2005), itself unique as noted above.

Even though it is a currently accepted subspecies (Wilson and Mittermeier 2011), COSEWIC (2011) renamed it the Eastern Migratory ecotype, DU4 (Fig. 2). COSEWIC (2011) also extended its range to the west of Hudson’s Bay and James Bay, based on small migratory/aggregated calving populations at the south end of James Bay in Ontario and Quebec (Abraham and Thompson 1998; Brown et al. 2003) and west into Manitoba (Pond et al. 2016). Genetic evidence supports this extension (Klütsch et al. 2016).

COSEWIC (2011) further designated a montane ecotype in the Torngat Mountains as DU12, based on ecological differences from the migratory ecotype (Bergerud 2000; Boulet et al. 2007; Couturier et al. 2010). Using satellite tracking and microsatellite markers, Boulet et al. (2007) found no significant genetic differentiation (pairwise F_ST_ all ≤ 0.5%) between migratory and montane herds, whereas woodland caribou of southern Quebec were significantly differentiated (F_ST_ = 1.8%–4.8%). Yannic et al. (2016) confirmed the lack of genetic distinction between Torngat and migratory Labrador caribou. Labrador caribou are also smaller than Quebec woodland caribou (Couturier et al. 2010). Since the Torngat Mountain montane ecotype DU12 is not genetically distinct at the subspecies level from other Labrador caribou, it needs no formal description as a subspecies and Allen’s type specimen designation from the Torngat Mountains (Fig. 2) remains valid for Labrador caribou, *R.c.caboti* Allen, 1914.

#### Newfoundland caribou

By contrast, the "totally different” (Geist 2007) Newfoundland caribou, *R.t.terraenovae* Allen, 1896, currently a valid subspecies (Wilson and Mittermeier 2011; Wilkerson et al. 2018), has distinctive pelage and antlers of the "classical ‘woodland form’ but of large dimensions and spreading” (Geist 1991). Analysis of mtDNA haplotypes confirmed that they are NAL woodland caribou that likely diverged during the LGM in a coastal refugium (Cronin et al. 2005; Wilkerson et al. 2018; Yannic et al. 2018). The name *R.caribouterraenovae* Allen, 1896 (Jacobi 1931; Anderson 1946) is available and appropriate, given its genetic proximity and phylogenetic descent from other woodland caribou.

## ﻿Discussion

### ﻿Invalid taxonomies

Some of the writers credited for caribou ecotypes’ first descriptions and names should not have been. Although Millais (1915b; 1915a), writing about trophy big game hunting in ‘The Gun at Home and Abroad’, produced a great travelogue for hunters, it is of little use to the taxonomist. He collected no type specimens, designated no type localities, and gave little or no description of anything except the horns and antlers. Outram Bangs, a member of a Boston nature club, named a couple dozen mammal species in club newsletters and pamphlets, including a two-page leaflet purporting to describe *R. terrænovæ* (Bangs 1896); he did not designate a type locality and his leaflet does not qualify as "published”. John J. Audubon was a great artist and an icon of American natural history, but he never saw a caribou, painted wildlife named by others, designated no type localities, and sent no specimens to any museum. His and John Bachman’s ‘The Quadrupeds of North America’ (Audubon and Bachman 1849), while a wonderful resource for artists and natural historians, does not quality as a taxonomic reference. I omit these from the synonymy (Suppl. material 1: Synonymy). Because Jacobi (1931) gave the same names to wild and domesticated reindeer from the same places, his descriptions of Eurasian *Rangifer* taxa are unreliable.

### ﻿What is a subspecies?

Traditional taxonomy, based on measurable morphological differences, usually in dental, cranial and skeletal characters, advanced greatly with the advent of phylogeny, or evolutionary history, with its emphasis on derived characters, especially those with functional significance. This is still the default paradigm for classifying fossils, except in rare cases where ancient DNA can be extracted. Geist (2007) said that Banfield’s (1961) principal error was using only skeletal (mainly cranial) metrics that, although seemingly objective, vary by age, gender, and condition of the specimens; he advocated using "nuptial” characteristics such as pelage colour patterns and antler shape, which are sexually selected and vary with mating systems, to diagnose subspecies: "These characteristics vary with the age of the males, are minimally affected by environment and are best expressed in old males at breeding time… selection for nuptial characteristics is done through female sexual selection” (Geist 2007).

The visual stimuli of pelage markings that differ by named subspecies (Geist 1998, 2007) are the "mate recognition system,” a central component of the "recognition concept” of the biological species definition (Patterson 1980). The pelage features that Geist (1998) asserts are sexually selected, highly conserved and variable among (but not within) subspecies are (1) the patterns of white and dark in the mane, (2) a light neck field that in some ecotypes extends onto the withers, (3) white socks or black hooves set off by a white band, (4) a light or dark rostrum, (5) a dark and/or a light lateral stripe, (6) a white belly that in some forms extents up the flanks and sides, (7) a secondary white rump patch, (8) white inside of the hind legs (and in some forms, the front legs) contrasting with brown or black frontal leg markings and (9) presence or absence of a light eye-ring.

Mating systems include, besides mate choice, male "fighting behaviour arising from a fundamental difference in mate-holding strategies”, female calving strategies, and anti-predator strategies (Geist 1991). They contrast markedly among the sedentary, dispersed forest types that use harem-holding or harem defence (cf. Hirotani 1989) and hide their calves in bogs, to the montane types with vertical migrations that also use harem-herding but disperse to calve on mountain ridges; and the tundra types that form tending bonds within large mating herds, synchronise calving, and form calf aggregations to minimise wolf predation (e.g., Bergerud 1974; Bergerud and Page 1987; Barrette and Vandal 1990; Mallory and Hillis 1998; Brown et al. 2003; Wittmer et al. 2006; Holand et al. 2007; Heatta 2009; Hegel et al. 2012; Flasko 2014; MacNearney et al. 2016; Weladji et al. 2017).

Butler (1986) showed that the social requirements of caribou females during the rut determines the mating strategies of males and, consequently, the form of male antlers.

Although sexually selected features are highly conserved, environment also drives both antlers (smaller to avoid entanglement in trees; shape and position of brow tines, "ice tines” in European parlance, to facilitate cratering in snow) and pelage (cryptic colouration: darker in forest, lighter on tundra). Forest reindeer and woodland caribou tend to have darker pelage, although the Altai reindeer (see above; Fig. 3), is sand- to red-coloured and not as dark as woodland caribou (Geist 1998). Woodland caribou are uniformly darker than barren-ground caribou, besides being up to twice their size. Geist (1991, 1998) illustrated the pelage patterns and antler types of mature males of most recognised subspecies: woodland, Newfoundland, Peary, barren-ground, Osborn’s, Labrador caribou, European tundra, and Svalbard reindeer; and he described diagnostic nuptial pelage and antler shapes of those that he did not illustrate: European forest, Novaya Zemlya, Siberian tundra, Altai, and Kamchatka reindeer. In considering how genetic data confirms or rejects traditional taxonomy, we should not overlook morphological features that have always guided diagnosis and that, in any case, remain essential for fossil forms (cf. Croitor 2018).

### ﻿Genetic distances

There are no generally accepted thresholds of genetic distance to distinguish species within a genus or subspecies within a species. Genetic distances comparing mtDNA sequences among cervid genera are generally 12%–18% (see Suppl. material 2: Genetic distance). Within cervid genera (i.e., between species), genetic distances in mtDNA sequences are generally around 3%–6% (e.g., Cronin 2003; Cai et al. 2016; Gutiérrez et al. 2017) and between subspecies around 1%–3% (e.g., Avise et al. 1998; Cai et al. 2016).

Nuclear microsatellite data give higher genetic distances, e.g., 16%–20% between pairs of white-tailed deer (*Odocoileusvirginianus*) subspecies (Rosa-Reyna et al. 2012). For comparison, genetic distance (Cavalli-Sforza and Edwards 1967) of microsatellite allele frequencies between pairs of Eurasian and North American tundra reindeer and caribou (that is, excluding Svalbard and Fennoscandian reindeer and Greenland and woodland caribou) ranged from 27.6% to 32.1% (see Suppl. material 2: Genetic distance).

Genetic differentiation, F_ST_, measures the variance in allele frequency among populations and describes the degree of genetic similarity among individuals within populations. F_ST_ and genetic distance measures are often highly correlated for a set of population or species pairs, the former usually being a little higher.

Genetic distances and F_ST_ data mentioned herein, and other data given in Suppl. material 2: Genetic distance, show that (1) within *Rangifer* exist populations currently identified as subspecies or below (e.g., ecotypes, populations) that are at least as distinct as species in other taxa, and (2) many previously named subspecies and some previously unidentified ecotypes are distinct enough to be recognised as subspecies, or even as full species.

## ﻿Taxonomic conclusions

The following names are available and should be used for ecotypes and phylogenetic clades of *Rangifer*:

### ﻿*Rangifergroenlandicus*

At a microsatellite genetic difference of F_ST_ = 44% from all other caribou (Yannic et al. 2013), the original name, *Rangifergroenlandicus* Gmelin, 1788 (Baird 1859) as Greenland caribou, type locality "Greenland”, distribution Greenland, is the appropriate name.

### ﻿*Rangiferplatyrhynchus*

The Svalbard Reindeer, as different from other Eurasian reindeer as Greenland caribou are from other North American caribou, should retain its original name, *Rangiferplatyrhynchus* Vrolik, 1829 (Sokolov 1932, 1963).

### ﻿*Rangifercaribou*

The genetic difference estimates between woodland caribou and barren-ground caribou, based on mtDNA, range from F_ST_ = 33% to > 50% (see Suppl. material 2: Genetic distance); this and lack of shared haplotypes except for minor, ancient introgression in some populations, is easily enough to separate them at the species level. Divergence time estimates of the split between forest (NAL) and barren-ground (BEL) clades range from 135,600 years ago during the penultimate (Illinoian) interstadial (Taylor et al. 2021) to a pre-Illinoian glacial period 300,000 years ago (Yannic et al. 2013). Moreover, woodland caribou may descend from extinct species of *Rangifer* in southern North America that never had contact with barren-ground caribou. This would not show up in genetic data except that they have unique haplotypes. In addition, they have a fundamentally different morphology (body size, antler size and formation including flattened vs. round tines, pelage, differences in dentition and in rostral structure), ecology, and behaviour. This clearly supports that the woodland caribou should be restored to species level, *Rangifercaribou* Gmelin, 1788 (Baird 1859).

Among *R.caribou* ecotypes and clades, the pattern of high differentiation of microsatellite allele frequencies and mtDNA haplotypes (relative to the barren-ground clade) results from isolation in at least four glacial refugia south of the ice sheets (Flagstad and Røed 2003; Cronin et al. 2005; Wilkerson et al. 2018). This justifies several subspecies. The following NAL lineage ecotypes, designated as distinct evolutionary units (DU—COSEWIC 2011) have available names:

- *R.c.caribou* Gmelin, 1788. Boreal woodland caribou DU6. Range restricted to mostly south of Labrador caribou with some overlap. Currently recognised (Wilson and Mittermeier 2011).
- *R.c.caboti* Allen, 1914 (Loughrey and Kelsall 1970), Labrador or Ungava caribou, Eastern Migratory caribou DU4, is currently recognised (Wilson and Mittermeier 2011).

- The Torngat Mountains montane caribou clade remains a valid ecotype.

- *R.c.terraenovae* Allen, 1896 (Jacobi 1931), Newfoundland caribou DU5. Currently recognised (Wilson and Mittermeier 2011).

The Atlantic-Gaspésie ecotype, DU11, is significantly differentiated genetically from other populations in Québec and throughout Canada. There is no available name.

Other woodland caribou clades across the boreal forest have considerable genetic distinction and may warrant subspecific designation but need more investigation.

### ﻿*Rangiferarcticus*

Mainland barren-ground caribou, currently recognised as *R.t.groenlandicus* (Wilson and Mittermeier 2011) is genetically and morphologically distinct from European tundra caribou and from woodland caribou. It clusters separately from Eurasian tundra reindeer and has pairwise genetic distances (microsatellite variation: Nei) of 20% to wild *R.t.tarandus* from Norway, but only 5% to Alaskan barren-ground caribou (Røed 2005). Cronin et al. (2006), using the Cavalli-Sforza and Edwards formula with microsatellite allele frequencies, put the genetic distance between Russian tundra reindeer and Canadian barren-ground caribou at 0.310. A microsatellite genetic distance of 20% to > 30% suggests specific differences between Eurasian tundra reindeer and North American barren-ground caribou.

However, *groenlandicus* cannot be applied to Canadian barren-ground caribou as a species name, as discussed above. *Rangiferarcticus*Richardson 1829, the first name applied to North American mainland barren-ground caribou (Ross 1861; Murray 1866) is the appropriate name.

Currently-accepted subspecies of *Rangiferarcticus*:

- *R.a.arcticus* Richardson, 1829, barren-ground caribou, DU3. Currently recognised as *R.t.groenlandicus* (Wilson and Mittermeier 2011) but restricted to tundra (summer) and boreal forests (winter) of mainland Canada and Alaska.
- *R.a.pearyi* Allen, 1908, Peary caribou, DU1. Currently recognised (Wilson and Mittermeier 2011).
- †*R.a.dawsoni* Seton-Thompson, 1899 (Jacobi 1931), the Queen Charlotte Islands caribou, DU12, currently recognised (Wilson and Mittermeier 2011), an *arcticus* subspecies of BEL lineage, as noted above (and see Suppl. material 2: Genetic distance). 1

- *R.a.osborni* Allen, 1902 (Murie 1935), Osborn’s caribou, DU7. Currently recognised (Wilson and Mittermeier 2011).

- The unnamed "woodland ecotype” of BEL lineage in the Mackenzie Valley (Polfus et al. 2017) warrants recognition as a unique ecotype of *R.a.osborni*.

Formerly recognised subspecies of *R.arcticus* that should be reinstated:

- *R.a.granti* Allen, 1902, Grant’s caribou. Restricted to Alaska Peninsula and archipelago, Alaska (Colson et al. 2014; Mager et al. 2014).
- *R.a.stonei* Allen, 1901 (Osgood 1909), Stone’s caribou, is available for interior Alaskan mountain caribou as a group that is coherently separable at the subspecies level from *granti*, *osborni*, and *arcticus*.

- *R.a.mcguirei* Figgins, 1919, Chisana mountain caribou. Original range provisionally designated as "the vicinity of the Alaska-Yukon boundary from the base of Mt. St. Elias northward” (Figgins 1919), restricted to that mapped by Mager et al. (2014). However, Murie (1935) gave good reasons for relegating *mcguirei* to a junior synonym of *R.a.stonei*.

- *R.a.fortidens* Hollister, 1912 (Jacobi 1931), DU8, Rocky Mountains caribou.
- *R.a.montanus* Seton-Thompson, 1899 (Jacobi 1931), Selkirk or mountain caribou, DU9, was formerly assigned to *R.arcticus* (Jacobi 1931; Anderson 1946), which its BEL lineage (see above) shows to have been correct.

Molecular analyses (see above) have revealed distinct subspecific clades of *R.arcticus* that have yet to be described:

- Dolphin and Union barren-ground caribou, DU2. Anderson’s (1913) brief, informal description of pelage and cranial differences from other barren-ground caribou, in view of later quantitative confirmation (e.g., Thomas and Everson 1982), would seem to warrant the new name of *R.arcticusandersoni* 1913. AMNH M-34433 would be a suitable neotype specimen, type locality "south side of Coronation Gulf”.
- Unnamed Baffin subspecies. Manseau et al. (2019) found it subspecifically distinct and Jenkins et al. (2018) recommended a DU designation. There is no available name.

### ﻿Eurasian Tundra reindeer

Eurasian reindeer diversity is clouded in the English literature because many geneticists labelled their samples as "*Rangifertarandus*” whether they were from domestic or wild types, or *R.t.tarandus*, *R.t.sibiricus* or *R.t.fennicus* (Western scholars seem not to have included *R.t.phylarchus*, *R.t.angustirostris*, and *R.t.valentinae* in their samples).

Domestic reindeer, a large, multi-faceted industry throughout Russia and Siberia, show little genetic exchange with wild reindeer and their population identities are mutually exclusive (e.g., Røed et al. 2008; Korolev et al. 2017).

- *R.t.tarandus* Trouessart, 1898, mountain reindeer, is restricted to Norway, Sweden, Finland and Russia (Murmansk).
- *R.t.sibiricus* von Schreber, 1784. Siberian reindeer, ranges from Arkhangelsk eastwards.

- If the Taymyr reindeer were to be separated from other Siberian reindeer on the basis of its migratory behaviour (Krivoshapkin 2016), as some have recommended (e.g., Michurin 1965; Pavlov et al. 1989), its name would be *R.t.taimyrensis* Michurin, 1965. However, its genetic separation (single-nucleotide polymorphisms, SNP) from the Yakutsk population of *R.t.sibiricus*, > 1,000 km to the east, is only F _ST_ = 0.5% (Kharzinova et al. 2020).

### ﻿Forest reindeer

Within Eurasian reindeer (Fig. 3), the most different in size and ecology from *R.t.tarandus* is the Finnish forest reindeer *R.t.fennicus*. Forest reindeer were probably isolated the longest of other forms and their apparent descent from the fossil reindeer *Cervusguettardi* Desmarest, 1820, precludes assignment to *R.tarandus*. Morphological, ecological, and genetic differences suggest *R.fennicus* Lönnberg, 1909 (Miller 1912a) as the appropriate name, with junior synonyms *silvicola*, *transuralensis*, and *dichotomus* (Fig. 3).

Subspecies are:

- the Finnish or western European forest reindeer *R.fennicusfennicus* Lönnberg, 1909. Range: forested parts of Finland and Murmansk/Kola Peninsula, Karelia, and Arkhangelsk in Russia.
- the Altai Mountains forest reindeer *R.f.valentinae* Flerov, 1933 (Sokolov 1937), its range restricted to the Altai Mountains.
- *R.t.* (*f*?) *angustirostris* awaits genetic sampling and phylogenetic analysis.

## Footnotes

† Extinct

## Supplementary materials

Supplementary material 1 Synonymy Data type docx file Explanation note Synonymy as it would have been before Ellerman and Morrison-Scott (1951) for Eurasia and before Banfield (1961) for North America. https://binary.pensoft.net/file/735594This dataset is made available under the Open Database License (http://opendatacommons.org/licenses/odbl/1.0/). The Open Database License (ODbL) is a license agreement intended to allow users to freely share, modify, and use this Dataset while maintaining this same freedom for others, provided that the original source and author(s) are credited.Lee E. Harding

Supplementary material 2 Genetic distance Data type docx file Explanation note Literature review on glacial periods and genetic distance relevant to *Rangifer*. https://binary.pensoft.net/file/735595This dataset is made available under the Open Database License (http://opendatacommons.org/licenses/odbl/1.0/). The Open Database License (ODbL) is a license agreement intended to allow users to freely share, modify, and use this Dataset while maintaining this same freedom for others, provided that the original source and author(s) are credited.Lee E. Harding
