# ﻿Corrigenda: Harding LE (2022) Available names for *Rangifer* (Mammalia, Artiodactyla, Cervidae) species and subspecies. ZooKeys 1119: 117–151. https://doi.org/10.3897/zookeys.1119.80233

**DOI:** 10.3897/zookeys.1122.94672

**Published:** 2022-09-28

**Authors:** Lee Harding

**Affiliations:** 1 Canadian Wildlife Service (retired), Delta, Canada Canadian Wildlife Service Delta Canada

As a result of copy-editing cut-and-paste errors, incorrect authorities were given in two cases.

The corrected authorities are:

*R*[*angifer*] *platyrhynchus* (Vrolich, 1828).

*Rangifergroenlandicus* (Borowski, 1780) as Greenland Caribou (Kellogg 1932). Linnaeus (1767) gave *groenlandicus* as a junior synonym for circumpolar *R.tarandus*.

The Taxonomic Conclusions were not affected and the Synonymy (suppl. material 1) is correct.
