# Supplementary material for: ﻿Available names for Rangifer (Mammalia, Artiodactyla, Cervidae) species and subspecies
Source: Zookeys. 2022 Aug 26;1119:117–51. doi: 10.3897/zookeys.1119.80233 (PMC9848878; doi:10.3897/zookeys.1119.80233)
Supplement: Supplementary material 1 — Synonymy [file zookeys-1119-117_article-80233__-s001.docx]

Supplementary file 1 for Harding, “Available Names for Rangifer (Mammalia, Artiodactyla, Cervidae) species and subspecies”

# Synonymy

Eurasian synonymy as it would have been before Ellerman and Morrison-Scott 1951

### *Rangifer* Smith, 1827

*Cervus* Linnaeus, 1758: 66. Type species *Cervus camelopardalis* L. 1758:66.

*Tarandus* Gmelin, 1788: 177. Replacement name.

***Rangifer*** Smith, 1827: 304. Opinion 91 of the International Commission on Zoological Nomenclature placed *Rangifer* Smith, 1827, with *Cervus tarandus* Linnaeus, 1758 as type species, on the official list of names, signifying these as the authorities for the genus and species, respectively (International Commission on Zoological Nomenclature 1958). Charles Hamilton Smith is often cited as “Hamilton-Smith” or “Hamilton Smith”.

*Procerus* de Serres, 1832: 34 Type species *P. cariboeus* de Serres by monotypy. Name preoccupied (Grubb 2000).

*Procervus* de Blainville, 1840: 392. Replacement name for *Procerus* de Serres (Grubb 2000).

*Achlis* Reichenbach, 1845: 12. Replacement name for *Procervu*s de Blainville. Type species *Cervus tarandus* L.

*Rangifer platyrhynchus* (Vrolik, 1829). Svalbard Reindeer

*Cervus (Tarandus) platyrhynchus* Vrolik, 1829: 160. Type locality Spitzbergen.

*Cervus tarandus forma spetsbergensis* Andersén, 1862: 457. Type locality not specified, location “Spetsberg” (Spitsbergen). Although most authors credit Vrolik (1829), for the first description, Andersén (1862) believed that Vrolik’s skull was from Norway.

*Rangifer arcticus* var. *spitzbergensis*: Murray 1866: 155. Name combination and justified emendation of spelling.

*Rangifer tarandus spitzbergensis*: Lydekker 1898: 41. Name combination.

*Rangifer spitzbergensis*: Camerano 1902: 159. Name combination.

*Rangifer platyrhynchus*: Miller Jr. 1912: 985. Name combination. Miller Jr. (1912a) restored Vrolik’s original species name; first use of current name.

*Rangifer tarandus platyrhynchus*: Lydekker 1915: 243. Name combination.

*Rangifer platyrhynchus*: Sokolov 1932: 55. Name combination.

Type specimen not known. Neotype no. M2625, Zoologisk Museum, Universitet I Oslo, Oslo, Norway (Banfield, 1963).

### *Rangifer tarandus* (Linnaeus, 1758). Tundra Reindeer

*Cervus tarandus* Linnaeus, 1758: 67. Linnaeus (1758) gave *Rangifer* as a synonym of the species name. Linnaeus (1767) cited Edwards’ (1743) description and extended the range of *Cervus tarandus* to “Habitat in Alpibus Europae, Asiae, Americae.”

*Tarandus rangifer* Gmelin, 1788: 177. Replacement name.

α Cerv[us]. *Tarandus Rangifer*: Kerr, 1792: 297. Unjustified emendation of spelling.

*Cervus tarandus*: Desmarest 1822: 431. Name combination.

*Tarandus lapponum* Billberg, 1827: 22. Renaming of *Rangifer*; replacement name.

*Tarandus borealis* Rüppell, 1842: 183. Replacement name.

*Rangifer tarandus*: Grey 1843: 181. Name combination; first use of current name.

*Tarandus rangifer*: Gray 1852: 189. Name combination.

*Tarandus furcifer* Baird, 1852: 109. Type locality “mountains of Swedish Lapland.”

*Rangifer tarandus* Trouessart, 1898: 887. Type locality fixed by Lönnberg (1909) as “Mountains of northwest Sweden (Särna and Idre Parishes, Dalarna Province).”

Type specimen not known.

#### Subspecies of *Rangifer tarandus*

*Rangifer tarandus* *tarandus* L. 1758. Mountain Reindeer

Rangifer tarandus typicus Lydekker, 1898: 38. “wild Scandinavian Reindeer”.

Rangifer tarandus var. cilindricornis Camerano, 1902: 167. Camerano (1902) used cilindricornis and compressicornis to distinguish the two general types of antler architecture in Rangifer, the former occupying typically open habitats and the latter occupying forest habitats. However, he did not consider antlers to be a reliable taxonomic character (Gippoliti and Jan 2018).

Rangifer tarandus tarandus: Lydekker 1915. Name combination and first use of current name.

Type specimen of subspecies as for species.

*Rangifer tarandus sibiricus* (von Schreber, 1784). Siberian Reindeer

*Cervus sibiricus* von Schreber, 1784: pl. 248c.

*Rangifer tarandus* var. *sibiricus*: Murray 1866: 153. Type locality “Siberia. … eastward of the River Lena.”

*Rangifer tarandus sibiricus*: Lydekker 1902: 361. First use of current name.

*Rangifer arcticus asiaticus* Jacobi, 1931: 85. Type locality “Kolyma River, Northeastern Siberia.” Renaming of *sibiricus* of Murray, new type locality (a domesticated form).

*Rangifer tarandus transuralensis* Hilzheimer, 1936: 155. Type locality Konda River, Tuymen, Western Siberia.

Type specimen of *sibiricus* unknown; however, Jacobi (1931) deposited a type specimen of “*asiaticus*” in the Museum of Leningrad (ZMASL), Buturlin coll. no. 240-1908.

†*Rangifer tarandus pearsoni* Lydekker, 1902. Novaya Zemlya Reindeer

## *Rangifer tarandus pearsoni* Lydekker, 1902: 361. Type locality “Island of Novaya Zemlya.”

*Rangifer tarandus tarandus*: Jacobi 1931: 64. Replacement name.

*Rangifer tarandus pearsoni*: Flerov 1933: 336. Name combination.

Type specimen “In the possession of H. J. Pearson, Esq., Bramcote, Nottinghamshire, England” (Flerov 1933: 332).

†*Rangifer tarandus setoni* Flerov, 1933. Seton’s or Saghalien Reindeer

Rangifer tarandus setoni Flerov, 1933: 337. Type locality “Saghalien” (Sakhalin Island). Flerov (1933) described Sakhalin Island reindeer as “Closely allied to the R. tarandus phylarchus by its cranial characters but well distinguished from all Palaearctic wild reindeer by the dark brown colour of the belly without whitish area” which makes it a forest reindeer. Ellerman and Morrison-Scott (1951) continued to recognize R. t. setoni, but Banfield (1961) considered it a junior synonym of R. t. fennicus. Although Flerov was describing wild reindeer, the island today has only domesticated reindeer.

Type specimen adult male; skin ZMASL no. 2673, skull no. 6348.

## *Rangifer fennicus* Lönnberg, 1909. Forest Reindeer

*Rangifer tarandus fennicus* Lönnberg, 1909: 10. Type locality “Torne District [in Enontekiö], Finnish Lappland.”

*Rangifer fennicus*: Miller Jr. 1912: 981. Replacement name.

Type specimen No. 4661, collection of mammals, NR, Stockholm, Sweden (Lydekker 1915). Banfield (1963) erroneously designated a holotype no. 809/1960, University of Helsinki, Helsinki, Finland.

## Subspecies of *Rangifer fennicus*

*Rangifer fennicus fennicus* Lönnberg, 1909 Finnish Forest Reindeer

*Rangifer fennicus fennicus* Lönnberg, 1909. Type locality as above.

*Rangifer fennicus valentinae* Flerov, 1933. Altai Forest Reindeer

*Rangifer tarandus valentinae* Flerov, 1933: 336. Type locality “Head of Chulyshman River, North-Eastern Altai, Siberia.”

*Rangifer fennicus valentinae*: Sokolov 1937. Sokolov (1937) included *valentinae* as a subspecies of *Rangifer fennicus*. Banfield (1961) considered it a junior synonym of *R. t. fennicus*.

*Rangifer tarandus* *dichotomus* Hilzheimer, 1936: 157. Type locality “Seitowski, Possad, near Orenburg” (USSR).

*Rangifer tarandus silvicola* Hilzheimer, 1936: 155. Type locality “Olnets Province, southern Karelia [Republic of Karelia]”.

Type specimen adult male; skin ZMASL no. 22599, skull no. 10214.

*Rangifer fennicus phylarchus* Hollister, 1912. Kamchatka Reindeer

*Rangifer phylarchus* Hollister, 1912: 6. Type locality “Southeastern Kamtchatka [Kamchatka].” Range included the coast of Okhotsk Sea and Amurland, but DNA analysis shows it restricted to the Kamchatka Peninsula (Rozhkov et al. 2020).

*Rangifer tarandus phylarchus*: Lydekker 1915: 245. Name combination.

*Rangifer phylarchus*: Jacobi 1931: 130. Name combination.

*Rangifer fennicus phylarchus*: Sokolov 1937: 64. Sokolov (1937) included *phylarchus* as a subspecies of *Rangifer fennicus*. Banfield (1961) considered it a junior synonym of *R. t. fennicus*. Geist (1998) considered it a subspecies of *R. arcticus*.

Type specimen USNM No. 21343.

*Rangifer fennicus angustirostris* Flerov, 1932. Narrow-nosed Reindeer

*Rangifer angustirostris* Flerov, 1932: 8, Bargusin Reindeer, Narrow-muzzled Reindeer. Type locality Bargusin Mountains, north-eastern coast of Lake Baikal. Flerov (1933) gave the type locality as “Highlands of Bargusin Mountains, between the rivers Bolshoi Chiverkuy and Bolshaya Bannaya, northeastern coast of Lake Baikal, Siberia.”

*Rangifer tarandus angustirostris*: Ellerman and Morrison-Scott 1951: 376. Ellerman and Morrison-Scott (1951) accepted *angustirostris* as a subspecies, *Rangifer tarandus angustirostris,* even though Sokolov (1937) had not. Banfield (1961) considered it a junior synonym of *R. t. fennicus*. Genetic data are not available to determine whether its affinity lies with *R. fennicus* or *R. tarandus* (Rozhkov et al. 2020). See main manuscript for a discussion.

Type specimen adult male; skin ZMASL no. 20412, skull no. 10253.

# North America: Synonymy as it would have been before Banfield 1961

† *Rangifer tarandus eogroenlandicus* Degerbøl, 1957. East Greenland Caribou

*Rangifer tarandus eogroenlandicus* Degerbøl, 1957: 1. Type locality “Hekla Havn, Danmarks ø. South of Milne Land at head of Scoresby Sound, East Greenland”. Probably a junior synonym for *R. a. pearyi*.

Type specimen not known.

*Rangifer groenlandicus* (Borowski, 1780). Greenland Caribou

*Capra groenlandicus* Edwards, 1743: 51. Type locality, Greenland. Edwards (1743) predates Linneaus (1758) and is not available for nomenclatural purposes. Edwards (1743) claimed to have seen a male specimen (“head of perfect horns…”) from Greenland and said that a Captain Craycott had brought a live pair from Greenland to England in 1738.

*Cervus grœnlandicus*: Brisson 1756: 88. Type locality “Greenland”. Brisson’s (1756) mammal names predate Linneaus (1758) and are not available for nomenclatural purposes.

*Cervus grœnlandicus*: Linnaeus 1767: 93. Synonym for *Cervus tarandus*.

*Cervus grönlandicus* Borowski, 1780: 72. Unjustified emendation to correct spelling. Type Locality “Greenland”.

*(Cervus tarandus) γ grœnlandicus*: Gmelin 1788: 177. Name combination; justified emendation of spelling.

*Rangifer grœnlandicus*: Baird 1859: 634. Replacement name.

*Rangifer Groënlandicus*: Ross 1862: 141. Unjustified emendation of spelling.

*Rangifer grœnlandicus*: Allen 1896: 234. Justified emendation of spelling.

*Rangifer tarandus groenlandicus*: Lydekker 1915: 256. Type locality as above. Name combination.

*Rangifer grœnlandicus*: Miller Jr. 1924: 492. Name combination.

*Rangifer tarandus groenlandicus*: Jacobi 1931: 69. Name combination.

*Rangifer arcticus groenlandicus*: Allen 1942: 305. Name combination.

*Rangifer tarandus groenlandicus*: Anderson 1946: 181. Anderson (1946) recognized Greenland caribou as distinct from *R. arcticus* and placed the former under the Eurasian form.

*Rangifer tarandus groenlandicus*: Flerov 1952: 246. Flerov (1952) recognised *groenlandicus* as Greenland caribou and *arcticus* as barren-ground caribou.

Neotype no. 8831, National Museum of Canada, Ottawa, Canada from Nugsuak Peninsula, west coast of Greenland (Banfield 1963).

*Rangifer arcticus* (Richardson, 1829). Barren-ground Caribou

*Cervus tarandus* var*. α arctica* Richardson, 1829: 341. Type locality “Fort Enterprise, Winter Lake, Mackenzie District, N.W.T., Canada” (Allen, 1908). See subspecies below.

*Cervus tarandus var. α arctica* King, 1836: 207 as Barren Ground caribou. Replacement name.

*Tarandus arcticus*: Baird 1852: 105. Name combination and justified emendation of spelling.

*Rangifer groenlandicus*: Baird 1859: 634 as Barren Ground Caribou. After justifying barren-ground caribou as a distinct species from Eurasian reindeer, and considering whether it should be named *arcticus* or *groenlandicus*, Baird (1859) used the name *R. groenlandicus* because he thought it had priority, although with misgivings: “It is a question, admitting this to be a distinct species, whether it should bear the name of *groenlandicus* or *arcticus*. Following the strict law of priority, however, I have retained the former, although objectionable on account of its local character.”

*Rangifer arcticus*: Ross 1861: 438. Name combination.

*Rangifer arctica*: Allen 1942: 583. Presumed mis-spelling of *arcticus*.

*Rangifer tarandus*: Ellerman and Morrison-Scott 1951: 375, “Reindeer, Caribou”. Replacement name applied to “Arctic regions of Old and New Worlds…Arctic regions of North America, Greenland included” (Ellerman and Morrison-Scott 1951). They did not, however, designate a subspecies for North America.

*Rangifer tarandus arcticus*: Flerov 1952: 246. Flerov (1952) recognized *arcticus* as distinct from *groenlandicus*.

Type specimen apparently lost: “Dr. King…after saying that his investigations and collections fully prove them distinct, regrets the loss of the latter…” (King 1836 quoted by Baird, 1859). However, Lydekker (1915) mentions two barren-ground caribou specimens in the British Museum: BMNH no. 51. “Skull and antlers. Arctic America. Purchased (Argent), 1851” and BMNH no. 55 (702, w) “Skeleton and antlers. Arctic America; collected by Dr. J. Rae. Purchased, 1855.” John Rae explored the Barrenlands and collected specimens from Hudson’s Bay to the Arctic coast near [Victoria Island](https://en.wikipedia.org/wiki/Victoria_Island_(Canada)) from 1848 to 1854.

Neotype no. 22066, National Museum of Canada, Ottawa, Canada (Banfield 1963).

#### Subspecies of *Rangifer arcticus (*Richardson, 1829)

*Rangifer arcticus arcticus* (Richardson, 1829). Barren-ground Caribou

*Cervus tarandus* var*. α arctica* Richardson, 1829: 241. Type locality as for species.

*Rangifer tarandus arcticus*: Lydekker 1898: 47. Name combination and justified emendation of spelling.

*Rangifer arcticus arcticus*: Miller Jr. 1924: 491. Name combination.

*Rangifer tarandus arcticus*: Flerov 1952: 246-247. Name combination.

Type specimen not known; see *R. arcticus* above.

† *Rangifer arcticus dawsoni* Seton-Thompson, 1899. Dawson’s Caribou

*Rangifer dawsoni* Seton-Thompson, 1900: 257. Type locality Graham Island, Queen Charlotte Islands.

*Rangifer tarandus dawsoni*: Lydekker 1915: 251. Name combination and first use of current name.

*Rangifer arcticus dawsoni*: Jacobi 1931: 95. Name combination.

*Rangifer tarandus dawsoni*: Flerov 1952: 247. Name combination.

*Rangifer dawsoni*: Cowan and Guiguet 1965: 383. Name combination.

*Rangifer tarandus dawsoni*: Hall and Kelson 1959: 1018. Name combination.

Type specimen BCPM No. 1483.

*Rangifer arcticus montanus* Seton-Thompson, 1899. Mountain Caribou

*Rangifer montanus* Seton-Thompson, 1899: 129. Type locality “… taken in the Illecillewaet watershed, near Revelstoke, Selkirk Range, B. C., in I889."

*Rangifer arcticus montanus*: Jabobi 1931. Name combination.

*Rangifer tarandus montanus*: Flerov 1952: 246-247. Name combination and first use of current name.

Type specimen NMC no. 232, a whole, mounted specimen.

***Rangifer arcticus osborni* Allen 1902, Osborn’s Caribou**

*Rangifer osborni* Allen, 1902: 149. Type locality Cassiar Mountains, British Columbia.

*Rangifer montanus osborni*: Osgood 1909: 74. Name combination.

*Rangifer arcticus osborni*: Murie 1935: 81. Name combination.

*Rangifer tarandus osborni*: Hall and Kelson 1959: 1020. Name combination and first use of current name.

Type specimen AMNH no. 15714.

*Rangifer arcticus stonei* Allen, 1901. Stone’s Caribou

*Rangifer stonei* Allen, 1901: 143. Type locality Kenai Peninsula, Alaska. Range “most of central and northern Alaska, excluding Alaska Peninsula and Unimak Island; also in western Yukon, Canada” (Murie 1935). Murie noted the absence of caribou on the Kenai Peninsula, where it was described, and explained it by describing long migrations in central Alaska through forested lands. “The Kenai Peninsula seems to be simply an overflow area that probably often received an influx of [Stone’s] caribou from unusual migratory movements of interior herds” (Murie 1935).

*Rangifer arcticus stonei*: Osgood 1909: 18. Name combination.

*Rangifer tarandus stonei*: Hall and Kelson 1959: 1020. Name combination.

*Rangifer excelsifrons* Hollister, 1912: 5. Type locality “Meade River, near Point Barrow, Alaska”. Type specimen U. S. National Museum No. 16755. Murie (1935) synonymised *excelsifrons* with *stonei* because the basis of Hollister’s description was a single, highly unusual skull. Syn. *stonei*.

*Rangifer mcguirei* Figgins, 1919: 1. Type locality Kletson Creek, a tributary of White River, Yukon, Canada. Holotype: DMNH 1846 (Jones 1994). Murie (1935) synonymised *mcguirei* with *stonei* because (see manuscript). Syn. *stonei*.

Type specimen AMNH no. 16701.

*Rangifer arcticus fortidens* Hollister, 1912. Rocky Mountain Caribou

*Rangifer fortidens* Hollister, 1912: 3. Type Locality “head of Moose Pass branch of the Smoky River, Alberta (north-east of Mount Robson)”.

*Rangifer tarandus fortidens*: Lydekker 1915: 251. Name combination.

*Rangifer fortidens*: Miller Jr. 1924. Name combination.

*Rangifer arcticus fortidens*: Jacobi 1931: 94. Name combination.

*Rangifer tarandus fortidens*: Hall and Kelson 1959: 1020. Name combination.

Type specimen USNM No. 174505.

*Rangifer arcticus granti* Allen, 1902. Grant’s Caribou

*Rangifer granti* Allen, 1902: 119. Type locality “Western end of Alaska Peninsula, opposite Popoff Island, Alaska”.

Rangifer tarandus granti: Lydekker 1915: 253. “Inhabits treeless districts of the Alaskan

Peninsula and some of the adjacent islands”.

*Rangifer granti*: Miller Jr. 1924: 492. Name combination.

*Rangifer arcticus granti*: Murie 1935: 74. Name combination. Murie (1935) placed all Alaskan caribou (35 specimens) under *R. arcticus* because their skeletal and skull measurements, dental characters, pelage features, size and antler types overlapped with barren-ground caribou (8 specimens from “northern Canada”, “an inadequate series of typical *arcticus*”), although he noted that the Alaskan animals were uniformly heavier. He determined, however, that in the Alaska Peninsula and Unimak Island caribou, “the antlers of *granti* appear to have a tendency toward wide divergence, are comparatively slender and light, usually without a great number of points; the pelage may average a little paler; and the animals in general probably are smaller than *stonei*” (Murie 1935).

Type specimen AMNH no. 17593.

*Rangifer arcticus pearyi* Allen, 1908. Ellesmere Land Caribou

*Rangifer pearyi* Allen, 1908: 409. Type locality “Ellesmere Land [Ellesmere Island], N. Lat. 79⁰.” Jacobi (1931) placed *R. pearyi* in the “arcticus group” as a distinct species; Anderson (1946) concurred.

*Rangifer arcticus pearyi*: Manning 1960: 47. Name combination. See text

*Rangifer tarandus pearyi*: Flerov 1952: 246. Name combination and first use of current name.

Type specimen AMNH no. 19231.

*Rangifer caribou* (Gmelin, 1788). Woodland Caribou

[*Cervus tarandus*] *γ caribou* Gmelin, 1788: 177. Gmelin (1788) did not designate a type locality, although Banfield (1961) attributes “Quebec, Province of Quebec, Canada” to Gmelin 1788. See subspecies below.

γ *Cerv. Tarandus Caribou*: Kerr 1792: 297. Unjustified emendation of spelling.

*Tarandus rangifer* Ogilby, 1836: 134. Replacement name.

*Rangifer tarandus* De Kay, 1842: 121. Replacement name for woodland caribou.

*Cervus hastalis* Agassiz, 1847: 187. Replacement name.

*Rangifer hastalis*: Baird 1852: 109. Name combination.

*Rangifer caribou*: Baird 1859: 633. Name combination. First available use of Rangifer *caribou* for woodland caribou.

*Rangifer tarandus caribou*: True 1885: 592. Name combination.

*Rangifer caribou*: Trouessart 1898: 888. Name combination.

Type specimen not designated; however, De Kay (1842) based his description on a “specimen in the cabinet of the Medical College at Albany [that] came from Nova-Scotia.”

## Subspecies of woodland caribou

*Rangifer caribou caribou* (Gmelin, 1788). Woodland Caribou

[*Cervus tarandus*] *caribou* Gmelin, 1788: 17.) (See above). Type Locality not given; amended to “eastern Canada” (Miller Jr. 1912b).

*Rangifer tarandus caribou*: True 1885: 111. Name combination.

*Rangifer caribou caribou*: Miller Jr. 1912: 392. Name combination for Eastern Woodland Caribou (as distinct from Western Woodland Caribou (see below).

*Rangifer tarandus caribou*: Lydekker 1915: 240. Name combination.

*Rangifer caribou caribou*: Allen 1942: 317. Name combination.

*Rangifer tarandus caribou*: Flerov 1952: 246-247. Name combination.

Type specimen not designated. Neotype no. 4800, National Museum of Canada, Ottawa, Canada (Banfield, 1963). After their Mackenzie River Expedition (1825–1827), of which John Richardson was an officer, Sir John Franklin gave (or sold) several specimens to the British Museum; although the museum’s records are vague, Lydekker (1915) mentions two mounted specimens of woodland caribou, both BMNH no. 702: “Skull and antlers. North America; collected by Sir John Franklin”, no date, and “Antlers, female. North America; same collector.”

*Cervus tarandus var. β., sylvestris* Richardson 1829: 250. Part, not *R. t. caribou*. Type locality not specified. Neotype locality: Southwestern shores of Hudson Bay by Miller Jr. 1912. Richardson (1829) gave its range as “extending at the distance of eighty or a hundred miles from the shores of Hudson's Bay, from Athapescow Lake [Lake Athabasca] to Lake Superior," i.e., the Canadian Shield portion of the boreal forest from Ontario to Alberta. Lydekker (1898) gave it as a junior synonym for *R. t. caribou*. Nomen dubium (see main manuscript).

*Cervus tarandus sylvestris*: Gilpin 1870: 58. Name combination; nomen dubium.

*Rangifer caribou sylvestri*s: Hollister 1912: 4. Name combination. Type locality amended to “southwestern shores of Hudson Bay”. Name combination; nomen dubium.

Type specimen not designated: “No specimen in collection” of the British Museum (Lydekker 1915).

*Rangifer caribou caboti* Allen 1914. Labrador Caribou

*Rangifer arcticus caboti* Allen, 1914: 104. Type locality “Thirty miles north of Nachvak [Torngat Mountains], northeast coast of Labrador”.

*Rangifer caboti*: Jacobi 1931: 108. Name combination.

*Rangifer tarandus caboti*: Flerov 1952: 246-247. Name combination.

*Rangifer caribou caboti*: Loughrey and Kelsall 1970. Name combination.

Type specimen MCZ No. 15,372.

*Rangifer caribou terraenovae* Allen 1896. Newfoundland Caribou

*Rangifer tarandus terrænovæ* Allen, 1896: 233. Type Locality “Grand Lake, Newfoundland”. Allen (1896) credited Bangs (1896) with the name and description in a leaflet distributed in November, 1896, 10 days before Allen’s paper was printed, which, however, did not meet the definition of a publication (see text). Anderson (1946) amended its range to Newfoundland.

*Rangifer terraenovae*: Trouessart 1898: 888. Name combination and justified emendation in spelling of *terrænovæ*.

*Rangifer caribou terranovae*: Jacobi 1931: 122. Name combination and unjustified emendation in spelling of terraenovae.

*Rangifer caribou terranovae*: Anderson 1946: 181. Name combination.

*Rangifer tarandus terranovae*: Flerov 1952: 246-247. Name combination.

*Rangifer tarandus terraenovae*: Hall and Kelson 1959: 1018. Name combination and justified emendation in spelling.

Type specimen MCZ B 3778 (Bangs 1896); AMNH 11775 (Allen 1896). Allen’s (1896) description was based on six specimens collected in 1895 and labelled, “*Rangifer tarandus terrænovæ*” in May 1896 as he was working on the description.

Abbreviations: **AMNH** American Museum of Natural History; **BCPM** British Columbia Provincial Museum (= **RBCM** Royal British Columbia Museum), **NHMUK** British Museum (Natural History) (originally BMNH), **DMNH** Denver Museum of Natural History, **MCZ** Museum of Comparative Zoology, **MSI** Museum of the Smithsonian Institution, **NMC** National Museum of Canada (originally the CGS Canadian Geological Survey Museum, now CMN Canadian Museum of Nature), **NR** Naturhistoriska Riksmuseet, **RSMNH** Royal Swedish Museum of Natural History, **USNM**, U. S. National Museum, **ZMASL** Zoological Museum of the Zoological Institute of the Russian Academy of Sciences (formerly the Zoological Museum of the Academy of Sciences), Leningrad.

† Extinct

### Literature cited in synonymy

Allen GM (1914) The barren-ground caribou of Labrador. Proceedings of the New England Zoölogical Club 4: 103-107.

Allen JA (1896) Description of new North American mammals. Bulletin of the American Museum of Natural History 8: 233-240, pls X and XI.

Allen JA (1908) Note on the type locality of *Rangifer arctica* (Richardson) Bulletin of the American Museum of Natural History 24: 583-584. http://hdl.handle.net/2246/526

Andersén CH (1862) Om Spetsbergsrenen, *Cervus tarandus, forma spetsbergensis*. Öfversigt af Kongl Vetenskaps-Akjademiens Forhandlingar 8: 457-461.

Anderson RM (1946) Catalogue of Canadian Recent mammals. National Museum of Canada Bulletin No. 102, Biological Series 31, Ottawa, Ontario, 238 pp.

Baird SF (1859) Mammals of North America: the description of species based chiefly on the collections in the museum of the Smithsonian Institution. J. B. Lippincott, Washington D.C., 764 pp.

Banfield AWF (1961) A revision of the reindeer and caribou, genus *Rangifer*. National Museum of Canada Bulletin 177, Biological Series No. 66, Ottawa, Ontario, 137 pp.

Banfield AWF (1963) A selection of neotypes for subspecies of *Rangifer tarandus* (Linné) (Mammalia-Cervidae). Contributions to Zoology 185: 60-71.

Bangs O (1896) Preliminary description of the Newfoundland Caribou. Manuscript, Boston, Massachusetts, 2 pp.

Brisson MJ (1756) Le Regnum Animale divisé en IX classes ou méthode contenant la division générale des Animaux en IX classes, & la division particulaire des deux premières classes, savoir de celle des Quadrupèdes & de celle des Cétacés, en Ordres, Sections, Genres & Espèces, vol. vi. Bausche, Paris/Leiden, 382 pp.

Camerano L (1902) Ricerche intorno alle renne delle isole Spitzberghe. Mémoires de l'Académie Royale des Sciences de Torino Series 2 51: 159-240.

De Kay JE (1842) Zoology of New-York or the New-York fauna comprising detailed descriptions of all the animals hitherto observed within the State of New York, with brief notices of those occasionally found near its borders, and accompanied by appropriate illustrations, vol. 2, part I Mammalia. D. Appleton & C[o.] / Wiley & Putnam, New York, 146 pp.

Edwards G (1743) A natural history of uncommon birds and of some other rare and undescribed animals quadrupedes, reptiles, fishes, insects &c exhibited in two Hundred and ten copper-plates, from designs copied immediately from nature, and curiously coloured after Life with a full and accurate description of each to which is added, a brief and general Idea of drawing and painting in water-colours; with Instructions for etching on copper with aqua fortis; likewise some thoughts on the passage of birds; and additions to many of the subjects described in this work. Vol. Part I, London, 248 pp. https://doi:10.5962/bhl.title.115782

Ellerman JE, Morrison-Scott TCS (1951) Checklist of Palaearctic and Indian mammals, 1758 to 1946. British Natural History Museum, London, 810 pp.

Flerov CC (1933) Review of the Palaearctic reindeer or caribou. Journal of Mammalogy 14: 328-338. https://doi:10.2307/1373952

Flerov CC (1952) Mammals: Musk deer and deer. In: Fauna of the USSR. Academy of Sciences, Moscow and Leningrad, USSR, 222-247.

Geist V (1998) Deer of the world: their evolution, behavior, and ecology. Stackpole Books, Mechanicsburg, Pennsylvania, 421 pp.

Gilpin JB (1870) On the Mammalia of Nova Scotia, No IV. Transactions of the Nova Scotian Institute of Natural Science 2: 58-69.

Gippoliti S, Jan R (2018) Lorenzo Camerano (1856-1917) and his contribution to large mammal phylogeny and taxonomy, with particular reference to the genera *Capra*, *Rupicapra* and *Rangifer*. Rendiconti Lincei Scienze Fisiche e Naturali 29: 443-451. https://doi:10.1007/s12210-018-0686-7

Gmelin JF (Ed) (1788) Systema Naturae Systema naturae per regna tria naturae: secundum classes, ordines, genera, species, cum characteribus, differentiis, synonymis, locis Edition 13. Vol. I, Emanuel Beer, Leipzig, Germany, 500 pp.

Grubb P (2000) Valid and invalid nomenclature of living and fossil deer, Cervidae. Acta theriologica 45: 289-307. <https://rcin.org.pl/ibs/publication/28962>

International Commission on Zoological Nomenclature (1958) Opinion 91 thirty-five generic names of mammals placed in the Official List of Generic Names In: Hemming F (Ed) Smithsonian Miscellaneous Collections, Opinions and declarations, Opinions rendered by the International Commission of Zoological Nomenclature, Opinions 91 TO 97. International Trust for Zoological Nomenclature, London, U.K., 337-338.

Jacobi A (1931) Das rentier: eine zoologische Monographie der Gattung *Rangifer*. Vol. Ergänzend band zu Band 96, Zoologischer Anzeiger, Leipzig, Germany, 204, 232 figs., 206 pls.

Jones C (1994) Type specimens of mammals in the Denver Museum of Natural History. Proceedings of the Denver Museum of Natural History Series 3 6: 1-4.

King R (1836) Narrative of a Journey to the Shores of the Arctic Ocean in 1833, 1834, and 1835. Vol. II, Richard Bentley, London, U.K., 321 pp.

Linnæi [Linnæus] C (1758) Systema Naturae Systema naturæ per regna tria naturæ: secundum classes, ordines, genera, species, cum characteribus, differentiis, synonymis, locis Edition 10 Tomus 1. Vol. 1, Laurentii Salvii, Holmæi [Stockholm], 824 pp.

Linné [Linnæus] Ca (1767) Systema naturae per regna tria naturae, secundum classes, ordines, genera, species, cum characteribus, differentiis, synonymis, locis Editio 12. Vol. Tomus I Editio duodecima, Vindobonae, Upsalla, Sweden, 1327 pp.

Lönnberg E (1909) Taxonomic notes about Palearctic reindeer. Arkiv för Zoologi 6: 1-17.

Loughrey AG, Kelsall JP (1970) The ecology and population dynamics of the barren-ground caribou in Canada. Ecology and Conservation Series Ecology of the Subarctic Regions Proceedings of the Helsinki Symposium. UNESCO, Paris, France, Helsinki, Finland, 275-280.

Lydekker R (1915) Catalogue of the ungulate mammals in the British Museum (Natural History): Artiodactyla, families Cervidæ (deer), Tragulidæ (chevrotains), Camelidæ (camels and llamas), Suidæ (pigs and peccaries), and Hippopotamidæ (hippopotamus). Vol. 4, Trustees of the British Museum, London, U.K., 385 pp.

Miller Jr. GS (1912a) Catalogue of the mammals of Western Europe (Europe exclusive of Russia) in the collection of the British Museum. British Museum (Natural History), London, U.K., 1019 pp.

Miller Jr. GS (1912b) List of North American land mammals in the United States National Museum, 1911. Smithsonian Institution United States National Museum Bulletin 79, Washington, D.C., 455 pp. https://[doi: 10.1126/science.37.951.453](https://doi.org/10.1126/science.37.951.453)

Murie OJ (1935) Alaska-Yukon caribou. Vol. 54, United States Department of Agriculture Bureau of Biological Survey, Washington D.C., 93 pp.

Murray A (1866) The geographic distribution of mammals. Day and Son, London, U.K., 420 pp.

Richardson J (1829) Fauna boreali-americana; or, the zoology of the northern parts of British America; containing descriptions of the objects of natural history collected on the late northern land expeditions, under command of Captain Sir John Franklin, R.N. Vol. 1 Quadrupeds, John Murray, London, U.K., 300 pp.

Rozhkov [Рожkов] ЮИ, Давыдов АВ, Моргунов НА, Осипов КИ, Новиков БВ, Майоров

АИ, Тинаев НИ, Чекалова ТМ, Якимов ОА (2020) ГЕНЕТИЧЕСКАЯ ДИФФЕРЕНЦИАЦИЯ СЕВЕРНОГО ОЛЕНЯ *Rangifer tarandus* L. ПО ПРОСТРАНСТВУ ЕВРАЗИИ В СВЯ- ЗИ С ОСОБЕННОСТЯМИ ЕГО ДЕЛЕНИЯ НА ПОДВИДЫ [Genetic differentiation of the reindeer *Rangifer tarandus* L. in Eurasia and its division into species]. КРОЛИКОВОДСТВО И ЗВЕРОВОДСТВО 2020: 23-36. https://doi:10.24411/0023-4885-2020-10203

Smith CH (1827) Order VII-Ruminantia. In: Cuvier G (Ed) Synopsis of the Species of the Class Mammalia, as arranged with reference to their organization by Cuvier and other naturalists with specific characters, synonyms, &c, &c. Goe. B. Whittaker, London, U.K., 296-376.

Sokolov [Соколов] II (1937) Sexual, age and racial variation of the skull of wild and domestic reindeer [in Russian]. Soviet Reindeer Industry 9: 1-102.

Thomas O (1911) The mammals of the Tenth Edition of Linnaeus; and attempt to fix the types of the genera and the exact bases and localities of the species. Proceedings of the Zoological Society of London 1911: 120-158.

Vrolik W (1829) Nieuwe Verhandlingen der erste Klasse van het Koninklijk-Nederlandsche Instituut 2: 160.
