# Supplementary material for: ﻿Available names for Rangifer (Mammalia, Artiodactyla, Cervidae) species and subspecies
Source: Zookeys. 2022 Aug 26;1119:117–51. doi: 10.3897/zookeys.1119.80233 (PMC9848878; doi:10.3897/zookeys.1119.80233)
Supplement: Supplementary material 2 — Genetic distance [file zookeys-1119-117_article-80233__-s002.docx]

Supplementary file 2 for Harding, “Available Names for *Rangifer* (Mammalia, Artiodactyla, Cervidae) species and subspecies”

**Genetic distance**

Since the discovery of polymerase chain reactions (PCR) to amplify DNA extraction in 1984, the discipline has exploded. There are ever more powerful statistical and other analytical techniques; DNA analysis has become more refined and less expensive, enabling researchers to sample more genetic markers of more specimens from more populations and to include nuclear and mtDNA in analyses of relatedness. Measures used to distinguish interspecific and subspecific populations include frequencies of alleles, shared vs. private haplotypes and haplogroups and other characteristics of mtDNA and nuclear DNA and genetic distance. Genetic distance describes the number of mutations or recombination events between pairs of chromosome DNA or mitochondrial DNA test results. Its basis is microsatellite allele or mtDNA haplotype frequency: populations with many similar alleles or haplotypes have small genetic distances. Genetic distances are expressed in centimorgans (cM) where 1 cM corresponds to a recombination frequency of 1%. Measures of genetic distance include Nei's standard genetic distance; Cavalli-Sforza chord distance; Reynolds, Weir, and Cockerham's genetic distance; and Fixation Index, F_ST_.

F_ST_, genetic differentiation, measures the variance in allele frequency among populations and describes the degree of genetic similarity among individuals within populations. A small F_ST_ means that allele frequencies within each population are very similar; a large F_ST_ means that allele frequencies are very different. F_ST_ and genetic distance measures are often highly correlated for a set of population or species pairs, F_ST_ usually being a little higher.

Although nuclear microsatellites have been widely applied to investigate genetic diversity and population structure, other measures, such as short tandem repeats (STR), simple sequence repeats (SSRs) and genome-wide single nucleotide polymorphism (SNP) markers, have gradually replaced them, due to their abundance, cost efficiency, and ease of automation (Ben et al. 2015).

Genetic distances among other cervid genera

Pitra et al. (2004) analysed the mtDNA cytochrome b gene to determine that, within the Cervinae, *Rangifer* groups as a sister clade to a clade of mule deer+white-tailed deer (*Odocoileus*) and three South American genera: brocket deer (*Mazama*), Pudu (*Pudu*), and swamp deer (*Blastoceros)*; these as a whole are sister to a clade of moose (*Alces*)+roe deer (*Capreolus*)+Chinese water deer (*Hydropotes*, which is currently placed in its own subfamily, Hydropotinae).

Gutiérrez et al. (2017) found genetic distances (percent sequence divergence in mtDNA using the Kimura two-parameter model, K2P) from *Rangifer* to other genera within Rangiferini to range from 0.058 to 0.097; other genus pairs were in similar ranges.

Shi et al. (2004) sequenced complete mtDNA genomes and used a Kimura 2 parameter (K2P) model to determine genetic distances (sequence divergence) among genera of Cervidae. They found genetic distances between *Alces* and *Cervus*, 0.166–0.178; between *Alces* and *Capreolus*, 0.147–0.150; between *Alces* and *Hydropotes*, 0.140; *Alces* and *Muntiacus* 0.138–0.148; *Alces* and *Odocoileus* between 0.119 (*O. hemionus*) and 0.131 (*O. virginianus*); *Alces* and *Rangifer* 0.132; *Rangifer* and *Capreolus* 0.128–0.138; *Rangifer* and *Cervus* 0.141–0.164; *Rangifer* and *Hydropotes* 0.134; *Rangifer* and *Muntiacus* 0.136-0.153; *Rangifer* and *Odocoileus* 0.126–0.138. Cai et al. (2016), using the K2P metric with mtDNA, found a mean distance between species in Chinese Cervidae of 3.4%, and between genera, 8.9%.

The average sequence divergence between Cronin et al.’s (2006) 15 reindeer mtDNA haplotypes and the white-tailed deer haplotype was 0.127 (12.7%) when used as an outgroup (this was less than that between woodland caribou and all other caribou and reindeer: see below).

In summary, genetic distances (sequence divergence in mtDNA) between genera within Cervidae range from approximately 6% to 18%.

The F_ST_ using genome-wide SNP between red deer *Cervus elaphus* and fallow deer, *Dama dama* was 0.74; a similar result was found for Nei’s genetic distance, 0.79 (Kasarda et al. 2017).

Using microsatellite SSRs, Hsiao et al. (2022) observed a shorter Nei’s genetic distance between sika and red deer (0.2663) than between sika deer and sambar, *Cervus unicolor* (0.5806) or red deer and sambar (0.4858). The pairwise F_ST_ results mirrored those of Nei’s genetic distance, with the F_ST_ value between red and sika deer (0.4148) being lower than that between red deer and sambar (0.6472) or sika and sambar (0.5260).

Using single nucleotide polymorphisms (SNPs), F_ST_ among genera in the cervid subfamily Odocoilinae were 0.90–0.99 and among Cervinae genera, 0.86; the equivalent genetic distance measures (Nei’s genetic distance) ranged from 0.02 between species (*C. elaphus* and *C. canadensis*) to 0.55 between genera (*Axis axis* and *Alces alces*) (Kasarda et al. 2017).

Genetic distances among species in other cervid genera

Between species pairs within genera of North American mammals, the average mtDNA genetic distance is 6.4% (Avise et al. 1998).

Within a species, Gutiérrez et al. (2017) found that the genetic distance (uncorrected genetic divergence using mtDNA cytochrome-*b* sequence data) between mule deer, *Odocoileus* *hemionus hemionus*, and a clade containing two other subspecies, Columbian black-tailed deer, *O. h. columbianus*,and *O. h. sitkensis*, averaged 6.2%, greatly exceeding mean pair-wise levels of divergences (all less than 3.6%) among the other 12 species (four genera of Odocoileini and Rangiferini) that they sampled. The authors concluded that “…reconciling taxonomy with phylogenetics would require elevating *columbianus* and *sitkensis* to species rank” (Gutiérrez et al. 2017).

Using single nucleotide polymorphisms (SNPs), an average F_ST_ value of 0.67 was found between species pairs of *Cervus nippon, C. canadensis*, and *C. elaphus* (Kasarda et al. 2017).

Genetic distances among subspecies in other cervid genera

In pairs of intraspecific phylogroups of mammals generally, genetic distance (mtDNA sequence divergence) ranged from 0.04 to 0.130 (4%–13%); among large mammals, the average was lower, 0.024 (2.4%) (Avise et al. 1998).

Cai et al. (2016) found a mean genetic distance of 1.3% within species (that is, between subspecies pairs).

In microsatellite data, between pairs of five subspecies white-tailed deer, *O. virginianus*, genetic distances ranged from 0.16 to 0.20 (16%–20%) (Rosa-Reyna et al. 2012). The F_ST_ metric gives intraspecific genetic differentiation in Cervidae from 0.116 to 0.223 (e.g., red deer from New Zealand and Germany—Židek et al. 2008; red brocket deer from isolated populations in Mexico—Serna-Lagunes et al. 2021).

Genetic distances among Rangifer subspecies

### Eurasian Reindeer

Genetic distance (Cavalli-Sforza and Edwards 1967) of microsatellite allele frequencies of woodland caribou were 0.307 to Alaskan reindeer, 0.319 to Russian reindeer, 0.327 to Scandinavian reindeer, 0.276 to Alaskan caribou, and 0.321 to barren-ground caribou (Cronin et al. 2006).

Within the Euro-Beringian lineage (BEL), in 16 nuclear microsatellite markers, the degree of genetic differentiation (F_ST_) among ecotypes ranged between 0.00 and 0.69 (mean F_ST_ = 0.11, 95% CI) (Yannic et al. 2018). The ecotypes from Greenland and Svalbard displayed the highest levels of genetic differentiation both between them (F_ST_ = 0.69, P < 0.001) and in comparison with other ecotypes (average F_ST_ Greenland = 0.44 ± 0.07, 95% CI; average F_ST_ Svalbard = 0.41 ± 0.06, 95% CI) (Yannic et al. 2018).

Within the five subpopulations of wild reindeer and the domestic reindeer on the Taymyr Peninsula, pairwise F_ST_ values and Nei’s genetic distances (DN) showed relatively higher genetic differentiation among the wild populations than among domestic reindeer (maximal F_ST_ and DN values were 0.046 vs. 0.023 and 0.353 vs. 0.151, respectively) (Kharzinova et al. 2016).

Forest reindeer samples from the eastern European plain (northern Karelia, Komi Republic, and Arkhangelsk) contain no mtDNA haplotypes in common with wild mountain reindeer from the central and southwestern Norway, but they do contain five haplotypes common to tundra reindeer from the European part of Russia, as well as to the reindeer from distant Taimyr population; genetic distances, however, between Norwegian and western Russian tundra reindeer were modest (0.4% except for Karelian forest reindeer, 0.9–1.6%) (Baranova et al. 2012). Further studies confirmed the phylogenetic status and common origin of wild tundra reindeer from the northeastern European plain of Russia and the Siberian tundra reindeer, *R. t. sibiricus* (Korolev et al. 2017). They also found a haplotype from an extinct forest ecotype (from the Kerzhenets River basin, Nizhny Novgorod) in three populations of extant taiga reindeer.

Kharzinova et al. (2018) confirmed her earlier finding (Kharzinova et al. 2016) of contrasting patterns in the genetic structure and a clear separation of the tundra and taiga reindeer, “consistent with their morphological and ecological differences”: pairwise F_ST_ values between wild tundra and taiga reindeer averaged 0.038 to 0.094.

Croitor’s (2010) palaeontological data supported earlier, genetic studies that showed that the

…genetic gap between the tundra [Eurasian] subspecies R. tarandus tarandus and the forest subspecies R. tarandus fennicus is not as large as in the case of North American forest caribou and barren ground caribou, [however] modern Eurasian tundra and forest subspecies have diphyletic origin from a smaller refugia [sic] in western Eurasia situated in close connection to the extensive ice sheet that covered Fennoscandia, and a larger refugia [sic] of Beringia.

*Rangifer t. fennicus*, at the time, included all Eurasian forest reindeer including *valentinae* and *phylarchus*, Banfield (1961) and others having synonymised them. Fennoscandian reindeer evolved in isolation from Eurasian tundra forms for long enough that they cluster separately (1,297 caribou genotyped at 16 nuclear microsatellite loci and 347 mtDNA cytochrome b sequences—Yannic et al. 2013).

Reindeer from valleys < 50 km apart in Svalbard showed weak (F_ST_ = 0.03), but significant differentiation likely due to genetic drift and philopatry (Côté et al. 2002).

### North American caribou

Cronin (2005) stated that,

The ecotypes of R. t. caribou each had unique mtDNA genotypes.... Two genotypes occurred only in Newfoundland (C1 and C2), 2 genotypes occurred only in Labrador (C3 and C4), 2 genotypes occurred only in Québec (C6 and C7), and 1 genotype occurred only in Alberta (C5). The lack of shared mtDNA genotypes among the ecotypes of R. t. caribou are reflected in the high pairwise F_ST_ values (Table 3) and an average F_ST_ of 0.5096.

The average pairwise F_ST_ estimates are relatively high between woodland caribou and central Canadian barren-ground caribou (mtDNA F_ST_ = 0.412–0.518; microsatellites F_ST =_ 0.076–0.122), and between woodland caribou and Alaskan barren-ground caribou (mtDNA F_ST_ = 0.281–0.355; microsatellites F_ST_ = 0.021–0.049); the latter did not share any haplotypes with *R. t. caribou* (Cronin et al. 2005). This basic difference has been confirmed by all other studies of North American caribou genetics (e.g., Zittlau 2009; Yannic et al. 2018).

With an expanded dataset, in their mtDNA MP consensus tree, Cronin et al. (2006) found that the woodland caribou (haplotype C6) grouped apart from all other reindeer (domesticated Russian and wild Norwegian and Svalbard), Alaskan caribou and Canadian (barren-ground) caribou. Genetic distances of microsatellite loci showed Canadian barren-ground caribou well distanced from Russian and Scandinavian reindeer (F_ST_ = 0.306–0.333) and closer to Alaskan barren-ground caribou—nominally, *granti*, but see text—(F_ST_ = 0.220). Their nuclear microsatellite UPGMA tree (18 loci) showed woodland caribou outside of the cluster of all other caribou and reindeer except for Scandinavian reindeer (which was influenced by the very divergent Svalbard reindeer), at a genetic distance of about 0.15 (Cronin et al. 2006). Cronin et al. (2006) concluded that, “The unique mtDNA haplotypes and differentiated microsatellite allele frequencies of woodland caribou and Scandinavian reindeer probably resulted from isolation south of the North American continental glaciers and in a northern European glacial refugium, respectively.”

The consensus tree of Cronin et al. (2006) includes a large clade containing all 15 of the reindeer haplotypes along with the wild barren-ground caribou haplotypes (C8, C12), contrasting with the woodland caribou haplotype (C6) that occurs in a separate clade. Cronin et al. (2006) found that between woodland caribou and all others (Canadian and Alaskan barren-ground, Russian tundra and “Scandinavian” (which included Norway+Svalbard), “…three haplotypes occur only in Scandinavian reindeer, resulting in significant differentiation between Scandinavia and Alaska (F_ST_ = 0.332) and Russia (F_ST_ = 0.249)”. Based on nuclear microsatellite loci, Cronin et al. (2006) reported:

Pairwise F_ST_ values indicate relatively low level of differentiation between Alaska and Russia (F_ST_ = 0.053, 95% CI 0.025–0.084) and more differentiation between Alaska and Scandinavia (F_ST_ = 0.100, 95% CI 0.059–0.144) and Russia and Scandinavia (F_ST_ = 0.121, 95% CI 0.084–0.158).

Within the North American lineage (NAL: woodland caribou), genetic differentiation ranged from,000 to 0.24 (mean F_ST_ = 0.08, 95% CI). The highest levels of genetic differentiation were found between the ecotypes from Newfoundland and the mainland (mean F_ST_ = 0.14, 95% CI) and with isolated ecotypes (e.g., average F_ST_ Gaspésie = 0.19 ± 0.06, 95% CI) (Yannic et al. 2018).

Peary caribou

In the Northwest Territories, McFarlane et al. (2014) found considerable genetic differentiation and distance were found among Arctic Islands Peary caribou species pairs (average F_ST_ = 0.040; average Nei = 0.155), but these were still less than among Peary caribou and mainland + Dolphin and Union barren-ground caribou (average F_ST_ = 0.063; average Nei = 0.331). Serrouya et al. (2012) found, using microsatellite markers of samples from all three North American subspecies and all western montane ecotypes, that at the broadest level of categorization, Peary caribou was the most distinct. It formed a clade distinct (F_ST_ = 0.07) from its nearest neighbour, the Dolphin and Union herd, now recognized as barren-ground caribou (COSEWIC 2004). A genetic differentiation of 7% would classify them as separate species in most interpretations. The genetic differentiation from *pearyi* to the recently extirpated Purcell subpopulation of mountain caribou in extreme south-eastern British Columbia, > 2,800 km away, was 22% (Serrouya et al. 2012), which would classify them as separate genera under most interpretations. See Jenkins et al. (2018) below regarding *pearyi*, Baffin Island, and barren-ground caribou.

Greenland caribou

Using microsatellite variation in 13 loci, Røed (2005) found high genetic distances between West Greenland caribou and all other types, including other Canadian barren-ground caribou (Nei: 0.69–2.21). His UPGMA cluster diagram based on nuclear microsatellite allele frequencies showed West Greenland *R. t. groenlandicus* clustering outside of every other subspecies. Yannic and colleagues (Yannic et al. 2013, 2018) confirmed this, finding that, based on both nuclear DNA and mtDNA haplotype lineages, the highest levels of genetic differentiation within the Beringian-Eurasian lineage were between Greenland and other BEL samples. They reported, based on 16 microsatellite loci, an average F_ST_ = 0.44 ± 0.07, 95% CI 0.42–0.46 between Greenland and other BEL reindeer and caribou (and even more to Svalbard reindeer, which also fall outside of the BEL clade: average F_ST_ = 0.69).

Grant’s caribou

In Cronin’s mtDNA dendrogram, the ecotypes of Alaska barren-ground caribou (formerly *R. t. granti)* and central Canadian barren-ground caribou each form separate clusters. Within the cluster of Alaska barren-ground caribou, the Central Arctic and Porcupine ecotypes occur together in a smaller cluster. Genetic differentiation between Alaska barren-ground caribou and central Canadian barren-ground caribou, based on microsatellite loci, ranged from F_ST_ 0.033 to 0.087; based on mtDNA, F_ST_ values ranged from 0.097 to 0.167 (Cronin et al. 2005).

Dawson caribou

Based on phylogenetic analysis of mtDNA, the insular *R. t. dawsoni* of Haida Gwai did not differ significantly from *R. t.* *granti* or *“R. t. caribou”* (Byun et al. 2002); however, their *“R. t. caribou”* samples were actually *R. t. osborni*, DU7 (cf. COSEWIC 2011), like *granti*, a BEL lineage (cf. McQuade Smith 2009; Polfus et al. 2017). Average intraspecific divergences (Byun et al. 2002) within *“caribou”* (= *osborni*) (0.018) and within *dawsoni* (0.014) were comparable to average intersubspecfic divergences between *granti* and *“caribou”* (= *osborni*) (0.016), between *granti* and *dawsoni* (0.019), and between *“caribou”* (= *osborni*) and *dawsoni* (0.020). This shows that *R. t. dawsoni* was a mountain caribou, not a woodland caribou, that became isolated on the islands.

Baffin Island caribou

Cronin et al. (2005) found that Baffin Island caribou share one haplotype with Labrador caribou and four with other Barren-land caribou. Genetic differentiation (mtDNA pairwise F_ST_) to Alaskan barren-ground caribou was 0.11.2–0.167; to central Canada barren-land caribou 0.049–0.077; to woodland caribou in Quebec and Alberta 0.298–0.427; to Labrador caribou 0.195; and to Newfoundland caribou 0.321. For 18 microsatellite loci, they found pairwise F_ST_ values of 0.058–0.087 to Alaskan barren-ground caribou, 0.089 to central Canada barren-ground caribou, 0.122–0.332 to woodland caribou, 0.127 to Labrador caribou, and 0.241 to Newfoundland caribou (Cronin et al. 2005).

Using 16 microsatellite loci, Jenkins et al. (2018) found pairwise F_ST_ values of 0.14 between Baffin Island caribou to two central Canada barren-ground ecotypes, 0.21–0.22 to Alaskan barren-ground caribou, and 0.24–0.28 to Peary caribou. They suggested that this difference and other genetic measures are evidence of intraspecific variation warrant a new Designatable Unit

(DU) (Jenkins et al. 2018). This implies at least a subspecies. There is no name available for a Baffin subspecies.

Woodland caribou, the NAL lineage

As noted above, in an expanded analysis of mtDNA and microsatellite loci, “woodland caribou” had a large genetic differentiation from Canadian barren-ground caribou (F_ST_ = 0.321; Cronin et al. 2006), which has been confirmed by others (e.g., Flagstad and Røed 2003; Zittlau 2004; Weckworth et al. 2018; Yannic et al. 2018; Manseau et al. 2019; Taylor et al. 2020). Cronin et al (2005) stated:

“Woodland caribou” [actually Labrador caribou, R. c. caboti] from the Eastern Migratory population DU4 (the George River herd), had relatively large genetic differentiation, using microsatellite DNA, to the central Canadian barren-ground caribou (F_ST_ = 0.099–0.104), but even higher to other woodland caribou (up to F_ST_ = 0.234), based on 18 microsatellite loci (Cronin et al. 2005). Measures of genetic differentiation were lower between that population and four Alaskan and Yukon populations of Grant’s caribou (F_ST_ = 0.147 to 0.199) than between the George River caribou and Val d’Or populations in boreal Québec (F_ST_ = 0.365). Differentiation was also high between the George River population and woodland caribou from Alberta (F_ST_ = 0.544). Similarly, higher genetic differentiation was detected between the George River population and other boreal caribou populations.

Boulet et al. (2007) showed that average genetic differentiation between seven eastern Canadian woodland caribou ecotypes was weak but significant (F_ST_ = 0.015). Although migratory (George River and Leaf River ecotypes) and a montane herd (*R. t. caboti*, Torngat Mountains), were not significantly distinct (F_ST_ all ≤ 0.005), they were significantly distinct from nonmigratory woodland caribou ecotypes of Quebec and Labrador (F_ST_ = 0.018–0.048) (Boulet et al. 2007). This was partly the basis of COSEWIC’s (2011) designation of the Eastern Migratory ecotype, DU4.

Mountain caribou

Allen (1900) described what he assumed were new specimens of *R. montanus* Seton-Thompson 1899 from the Cassiar Mountains in northern British Columbia; but in 1902 he received additional samples of *R. montanus* from south-eastern British Columbia, as well as a new series from southern Yukon, compared them and, realizing they were morphologically distinct, corrected himself, naming the Cassiar specimens *R. osborni* (Allen 1902).

Serrouya et al. (2012) found that:

...the genetic distance of the Purcell subpopulation to its neighbours < 250 km away (F_ST_ = 0.15) was greater than between some of the tundra ⁄ taiga ecotypes that were separated by > 1000 km (i.e., Peary to Qamanirjuaq F_ST_ = 0.07; Fig. 1). Even the directly adjacent deep-snow mountain subpopulations of Columbia North and Columbia South (5 km of separation, F_ST_ = 0.04) were more genetically distinct than large migratory ecotypes separated by almost 1000 km (e.g., Qamanirjuaq vs. Dolphin & Union, F_ST_ = 0.02). Furthermore, the difference between Columbia North and Columbia South was of the same magnitude as differences between subspecies (e.g., Graham [an R. t. osborni herd] vs. Qamanirjuaq; F_ST_ = 0.04; Fig. 1). Similarly, the genetic distance between Wells Gray and Columbia North subpopulations, which live as close as 5 km apart, was large relative to differences among ecotypes and subspecies. Populations south of the North Thompson Valley were relatively distinct from those to the north (F_ST_ = 0.04).

They attributed these differences more to genetic drift of subpopulations separated by major rivers, than to different evolutionary history (Serrouya et al. 2012).

Weckworth et al. (2012) analysed the mtDNA control region of 709 North American caribou and found that, of 151 haplotypes, 134 were unique to a subpopulation and “most haplotypes [were] endemic to one of the specific caribou types; 15, 10, 24, 29 and 56 haplotypes were unique to the Southern Mountain, Boreal, Northern Mountain, Barren-ground and Alaska caribou types, respectively”.

Weckworth et al. (2012) used a Kimura 3-parameter model to calculate “…pairwise $\Phi$ST distance matrix [their Table S1, Supporting information] that reflects evolutionary relationships between populations” based on mtDNA, and an F_ST_ matrix using nuclear microsatellite data [their Table S2]. They tested recognized western Canadian subspecies and ecotypes and other models of relatedness, but their study pre-dated COSEWIC’s (2011) recognition of the Central Mountain DU8 population as distinct, and they included it with “Southern Mountain” DU9 populations, of which they had only two specimens. They confirmed previous findings of a clear split between barren-ground BEL and woodland caribou NAL, clustered Osborn’s and Alaska barren-ground caribou with Canadian mainland barren-ground caribou, confirmed McDevitt et al.’s (2009) conclusion that the Central Mountain caribou are essentially hybrids between Boreal woodland and Mountain caribou DU9, and separated these from other boreal woodland caribou.

Weckworth et al. (2012) found a wide range of genetic differentiation (microsatellite F_ST_) from F_ST_ = 0.020 to 0.20 within what we now recognize as the Central Mountain population DU8 (originally *R. fortidens* Hollister 1912). Incredibly, the adjacent Jasper National Park (JNP) and extirpated Banff National Park (BNP) subpopulations differed by F_ST_ = 0.20; BNP may not have interbred with woodland caribou in ancient times to the same extent as JNP (cf. McDevitt et al., 2007) and is closer geographically to the Columbia North herd. BNP differed even more dramatically from the Slave Lake herd of Boreal (DU6) woodland caribou at F_ST_ = 0.32. Parsnip and Columbia North ecotypes, both now recognized as the Southern Mountain population DU9, differed from each other (F_ST_ = 0.070); they were well-differentiated from the BNP subpopulation (F_ST_ = 0.19 and 0.15, respectively) but less so (0.021 to 0.076) from other Central Population subpopulations. Between Central Mountain DU8 and the boreal woodland DU6 subpopulations, F_ST_ values ranged from F_ST_ = 0.033 to 0.152, the highest being Lesser Slave Lake in Alberta vs. Kennedy in British Columbia. These populations are separated by the Peace River, perhaps echoing Serrouya et al.’s (2012) conclusions.

The three Osborn’s samples hardly varied (F_ST_ = 0.003–0.035) and these were close to Alaskan BEL lineage barren-ground caribou (F_ST_ = 0.010–0.048), about the same range as to Canadian barren-ground caribou (F_ST_ = 0.010–0.036) confirming the same BEL lineage of all three ecotypes (Weckworth et al. 2012). Taylor et al. (2020) further confirmed the BEL lineage and added detail about divergence times and sub-lineages.

Within the Central Mountain DU8 population, F_ST_ ranged from 0.021 for the geographically closest subpopulations, Narraway-Redrock/Prairie Creek, to 0.182 for the farthest apart, Narraway-Banff (Weckworth et al. 2012).

Some examples of genetic differentiation between and within caribou populations are given in Table 1.

**Table 1.** Genetic distance (microsatellite F_ST_) among caribou populations.

| **Pairwise** | **Taxonomic level** | **F_ST_** | **Distance* (km)** | **Source** |
| --- | --- | --- | --- | --- |
| Purcell subpopulation-its neighbours | Subpopulations (Mountain) | 0.15 | < 250 | (Serrouya et al. 2012) |
| Columbia North–Columbia South | Subpopulations (Mountain) | 0.039 | 5 km | (Serrouya et al. 2012) |
| Wells Gray-Columbia North | Subpopulations (Mountain) | 0.041 | ≥ 5 km | (Serrouya et al. 2012) |
| South of the North Thompson Valley-north of it | Subpopulations (Mountain) | 0.04 | 5 km | (Serrouya et al. 2012) |
| North and south of Peace River | Populations (Boreal), mean of 6 species pairs | 0.059 | 5 km | (Serrouya et al. 2012) |
| Parsnip–Columbia North | Subpopulations (Mountain) | 0.070 | 475 km | (Weckworth et al. 2012) |
| Parsnip–Banff | Populations (Mountain–Rocky Mt.) | 0.19 | 550 km | (Weckworth et al. 2012) |
| Columbia North–Banff | Populations (Mountain–Rocky Mt.) | 0.15 | 100 km | (Weckworth et al. 2012) |
| Within Osborn’s | Subpopulations of *R. t. osborni* | 0.003–0.035 | various | (Weckworth et al. 2012) |
| Osborn’s–Alaskan | subspecies (*R. t. osborni–R. t. [granti*] *groenlandicus*) | 0.010–0.048 |  | (Weckworth et al. 2012) |
| Osborn’s–barren-ground | Subspecies (*R. t. osborni–R. t. groenlandicus*) | 0.010–0.036 |  | (Weckworth et al. 2012) |
| Narraway-Redrock Prairie Creek | Subpopulations (Rocky Mountain) | 0.021 | adjacent | (Weckworth et al. 2012) |
| Narraway-Banff | Subpopulations (Rocky Mountain) | 0.182 | 400 km | (Weckworth et al. 2012) |
| Rocky Mountain Caribou | Subpopulations (Rocky Mountain) | 0.021–0.182 | adjacent–650 km | (Weckworth et al. 2012) |
| Dolphin & Union-Qamanirjuaq | Populations (Barren-ground) | 0.02 | < 1,000 | (Serrouya et al. 2012) |
| Barren-ground– woodland | Subspecies (Slave Lake boreal *R. t. caribou* and *R. t. granti*) | 0.124–0.146 |  | (Weckworth et al. 2012) |
| Barren-ground– woodland | Subspecies *R. t. caribou* vs. *R. t. groenlandicus* | 0.3339 |  | (Cronin et al. 2005) |
| Peary–barren-ground (Qamanirjuaq) | Subspecies (*R. t. pearyi* and *R. t. groenlandicus*) | 0.07 | > 1,000 | (Serrouya et al. 2012) |
| Peary–southern mountain | Subspecies (*R. t. pearyi* and *R. t. caribou*, S. mt. ecotype) | 0.22 | > 2 800 km | (Serrouya et al. 2012) |

* Distance between population centres

# Literature

Allen JA (1900) The mountain caribou of northern British Columbia. Bulletin of the American Museum of Natural History 13: 1-18.

Allen JA (1902) Description of a new caribou from northern British Columbia and remarks on *Rangifer montanus*. Bulletin of the American Museum of Natural History 16: 149-158. <http://hdl.handle.net/2246/744>

Avise JC, Walker D, Johns GC (1998) Speciation durations and Pleistocene effects on vertebrate phylogeography. Proceedings of the Royal Society of London Series B: Biological Sciences 265: 1707-1712. https://doi:10.1098/rspb.1998.0492

Banfield AWF (1961) A revision of the reindeer and caribou, genus *Rangifer*. National Museum of Canada Bulletin 177, Biological Series No. 66, Ottawa, Ontario, 137 pp.

Baranova [Баранова] AI, Kholodova MV, Davydov AV, Rozhkov II (2012) Polymorphism of the mtDNA control region in wild reindeer *Rangifer tarandus* (Mammalia: Artiodactyla) from the European part of Russia. Russian Journal of Genetics 48: 939-944.

Ben JS, Boussaha M, Ben MM, Lee JH, Lee SH (2015) Genome-wide insights into population structure and genetic history of Tunisian local cattle using the illumina bovinesnp50 beadchip. BMC Genomics 16: 677. https://doi:pmid:26338661

Boulet M, Couturier S, Côte SD, Otto RD, Bernatchez L (2007) Integrative use of spatial, genetic, and demographic analyses for investigating genetic connectivity between migratory, montane, and sedentary caribou herds. Molecular Ecology 16: 4223-4240. <https://doi.org/10.1111/j.1365-294X.2007.03476.x>

Byun SA, Koop BF, Reimchen TE (2002) Evolution of the Dawson caribou (*Rangifer tarandus dawsoni)*. Canadian Journal of Zoology 80: 956-960. <https://doi.org/10.1139/z02-062>

Cai Y, Zhang L, Wang Y, Liu Q, Shui Q, Yue B, Zhang Z, Li J (2016) Identification of deer species (Cervidae, Cetartiodactyla) in China using mitochondrial cytochrome c oxidase subunit I (mtDNA COI). Mitochondrial DNA Part A 27: 4240-4243. https://doi:10.3109/19401736.2014.1003919

Cavalli-Sforza LL, Edwards AWF (1967) Phylogenetic analysis: Models and estimation procedures. American Journal of Human Genetics 19: 233–257. PMCID: PMC1706274

COSEWIC (2004) COSEWIC assessment and update status report on the Peary caribou *Rangifer tarandus peary*i and the barren-ground caribou *Rangifer tarandus groenlandicus* (Dolphin and Union population) in Canada. Committee on the Status of Endangered Wildlife in Canada (COSEWIC), https://wildlife-species.canada.ca/species-risk-registry/virtual_sara/files/cosewic/sr_peary_caribou_e.pdf, Ottawa, Ontario, 91 pp.

COSEWIC (2011) Designatable units for caribou (*Rangifer tarandus*) in Canada. Committee on the Status of Endangered Wildlife in Canada (COSEWIC), Ottawa, Ontario, 88 pp. http://www.cosewic.gc.ca/

Côté SD, Dallas JF, Marshall F, Irvine RJ, Langvatn R, Albon SD (2002) Microsatellite DNA evidence for genetic drift and philopatry in Svalbard reindeer. Molecular Ecology 11: 1923-1930. <https://doi.org/10.1046/j.1365-294X.2002.01582.x>

Cronin MA, MacNeil MD, Patton JC (2005) Variation in mitochondrial DNA and microsatellite DNA in caribou (*Rangifer tarandus*) in North America. Journal of Mammalogy 86: 495-505. [https://doi.org/10.1644/1545-1542(2005)86[495:VIMDAM]2.0.CO;2](https://doi.org/10.1644/1545-1542(2005)86%5b495:VIMDAM%5d2.0.CO;2)

Cronin MA, MacNeil MD, Patton JC (2006) Mitochondrial DNA and microsatellite DNA variation in domestic reindeer (*Rangifer tarandus tarandus*) and relationships with wild caribou (*Rangifer tarandus granti,* *Rangifer tarandus groenlandicus*, and *Rangifer tarandus caribou*). Journal of Heredity 97: 525-530. https://doi:10.1093/jhered/esl012

Flagstad O, Røed KH (2003) Refugial origins of reindeer (*Rangifer tarandus* L*.*) inferred from mitochondrial DNA sequences. Evolution 57: 658-670. <https://doi.org/10.1111/j.0014-3820.2003.tb01557.x>

Gutiérrez EE, Helgen KM, McDonough MM, Bauer F, Hawkins M, Escobedo-Morales LA, Patterson BD, Maldonado JE (2017) A gene-tree test of the traditional taxonomy of American deer: the importance of voucher specimens, geographic data, and dense sampling. ZooKeys: 87-131. https://doi:10.3897/zookeys.697.15124

Hsiao C, Lin H-H, Kang S-R, Hung C-Y, Sun P-Y, Yu C-C, Toh K-L, Yu P-J, Ju Y-T (2022) Development of 16 novel EST-SSR markers for species identification and cross-genus amplification in sambar, sika, and red deer. PLOS ONE 17: e0265311. https://doi:10.1371/journal.pone.0265311

Jenkins DA, Yannic G, Schaefer JA, Conolly J, Lecomte N (2018) Population structure of caribou in an ice-bound archipelago. Diversity and Distributions 24: 1092-1108. <https://doi.org/10.1111/ddi.12748>

Kasarda R, Moravčíková N, Trakovická A, Krupová Z, Kadlečík O (2017) Genomic variation across cervid species in respect to the estimation of red deer diversity. Acta Veterinaria 67: 43-56. https://doi:10.1515/acve-2017-0005

Kharzinova [Харзинова] VR, Dotsev AV, Deniskova TE, Solovieva AD, Fedorov VI, Layshev KA, Romanenko TM, Okhlopkov IM, Wimmers K, Reyer H (2018) Genetic diversity and population structure of domestic and wild reindeer (*Rangifer tarandus* L. 1758): A novel approach using BovineHD BeadChip. PLOS ONE 13: e0207944. https://doi:10.1371/journal.pone.0207944

Kharzinova [Харзинова] VR, Dotsev AV, Kramarenko AS, Layshev KA, Romanenko TM, Solov'eva AD, Deniskova TE, Kostyunina OV, Brem G, Zinovieva NA (2016) Study of the allele pool and the degree of genetic introgression of semi-domesticated and wild populations of reindeer (*Rangifer tarandus* L., 1758) using microsatellites. Sel’Skokhozyaistevennaya Biologiya [Agricultural Biology] 51: 811-823. https://doi:10.3390/ani10081309

Korolev A, Mamontov V, Kholodova M, Baranova A, Shadrin D, Poroshin E, Efimov V, Kochanov S (2017) Polymorphism of the mtDNA control region in Reindeer (*Rangifer tarandus*) from the mainland of the Northeastern part of European Russia. Biology bulletin 44: 882-893.https://doi.org/10.1134/S1062359017080106

Manseau M, Horn R, Taylor R, Wilson P (2019) A genomic refinement of Banfield’s 1961 revision of *Rangifer* for North American caribou. In: Skarin H (Ed) 15th International Arctic Ungulate Conference 12-16 August 2019. Jokkmokk, Sweden, 1-20.

McDevitt AD, Mariani S, Hebblewhite M, Decesare NJ, Morgantini L, Seip DR, Weckworth BV, Musiani M (2009) Survival in the Rockies of an endangered hybrid swarm from diverged caribou (*Rangifer tarandus*) lineages. Molecular Ecology 18: 665-679. https://doi: [10.1111/j.1365-294X.2008.04050.x](https://doi.org/10.1111/j.1365-294x.2008.04050.x)

McFarlane K, Miller FL, Barry SJ, Wilson GA (2014) An enigmatic group of arctic island caribou and the potential implications for conservation of biodiversity. Rangifer 34: 73-94. <https://doi.org/10.7557/2.34.1.2953>

McQuade Smith KA (2009) Investigating the genetic component to geographical variation in behaviour and metabolism in temperate mammals. M.Sc. thesis, Trent University, Peterborough, Ontario, 154 pp.

Pitra C, Fickel J, Meijaard E, Groves C (2004) Evolution and phylogeny of old world deer. Molecular Phylogenetics and Evolution 33: 880-895. <https://doi.org/10.1016/j.ympev.2004.07.013>

Polfus JL, Manseau M, Klütsch CFC, Simmons D, Wilson PJ (2017) Ancient diversification in glacial refugia leads to intraspecific diversity in a Holarctic mammal. Journal of Biogeography 44: 386-396. <https://doi.org/10.1111/jbi.12918>

Røed KH (2005) Refugial origin and postglacial colonization of holarctic reindeer and caribou. Rangifer 25: 19-30. https://doi:[10.7557/2.25.1.334](http://dx.doi.org/10.7557/2.25.1.334)

Rosa-Reyna XFDL, Calderón-Lobato RD, Parra-Bracamonte GM, Sifuentes-Rincón AM, DeYoung RW, León FJG-D, Arellano-Vera W (2012) Genetic diversity and structure among subspecies of white-tailed deer in Mexico. Journal of Mammalogy 93: 1158-1168. <https://doi.org/10.1644/11-MAMM-A-212.2>

Serna-Lagunes R, Romero-Ramos DK, Delfín-Alfonso CA, Salazar-Ortiz J (2021) Phylogeography of the Central American red brocket deer, *Mazama temama* (Artiodactyla, Cervidae) in southeastern Mexico. Neotropical Biology and Conservation 16: 369-382. https://doi:10.3897/neotropical.16.e58110

Serrouya R, Paetkau D, McLellan BN, Boutin S, Campbell M, Jenkins DA (2012) Population size and major valleys explain microsatellite variation better than taxonomic units for caribou in western Canada. Molecular Ecology 21: 2588-2601. <https://doi.org/10.1111/j.1365-294X.2012.05570.x>

Shi Y-F, Shan X-N, LI J, Shi T-Y, Zheng A-L (2004) Sequence analysis and phylogeny of deer (Cervidae) mtDNA control regions. Acta Genetica Sinica 31: 395-402.

Taylor R, Manseau M, Horn R, Keobouasone S, Golding B, Wilson P (2020) The role of introgression and ecotypic parallelism in delineating intra-specific conservation units. Molecular Ecology 29: 2793-2809. https://doi:10.1111/mec.15522

Weckworth BV, Hebblewhite M, Mariani S, Musiani M (2018) Lines on a map: conservation units, meta-population dynamics, and recovery of woodland caribou in Canada. Ecosphere 9: e02323. https://doi:10.1002/ecs2.2323

Weckworth BV, Musiani M, McDevitt AD, Hebblewhite M, Mariani S (2012) Reconstruction of caribou evolutionary history in western North America and its implications for conservation. Molecular Ecology 21: 3610-3624. https://doi: 10.1111/j.1365-294X.2012.0562

Yannic G, Ortego J, Pellissier L, Lecomte N, Bernatchez L, Côté SD (2018) Linking genetic and ecological differentiation in an ungulate with a circumpolar distribution. Ecography 41: 922-937. https://doi: 10.1111/e c og.02995

Yannic G, Pellissier L, Ortego J, Lecomte N, Couturier S, Cuyler C, Dussault C, Hundertmark KJ, Irvine RJ, Jenkins DA, Kolpashikov L, Mager K, Musiani M, Parker KL, Røed KH, Sipko T, Þórisson SG, V.Weckworth B, Guisan A, Bernatchez L, Côté SD (2013) Genetic diversity in caribou linked to past and future climate change. Nature Climate Change 4: 132-137. https://doi.[10.1038/nclimate2074](http://dx.doi.org/10.1038/nclimate2074)

Židek R, Pokorád J, Bandry Ľ (2008) Biodiversity in deer population observed by microsatellite markers Journal of Agrobiology 25: 113-115.

Zittlau KA (2004) Population genetic analyses of North American caribou (*Rangifer tarandus*). Ph.D. dissertation, University of Alberta, Edmonton, Alberta, 187 pp.

Zittlau KA (2009) Genetic diversity among woodland and Grant's caribou herds. In: McFarlane K, Gunn A, Strobeck C (Eds) Proceedings from the Caribou Genetics and Relationships Workshop, March 8-9, 2003. Department of Natural Resources and Environment, Government of the Northwest Territories Manuscript Report No. 183, Edmonton, Alberta, 147-164.
